# A *Sox2* enhancer cluster regulates region-specific neural fates from mouse embryonic stem cells

**DOI:** 10.1093/g3journal/jkaf012

**Published:** 2025-01-24

**Authors:** Ian C Tobias, Sakthi D Moorthy, Virlana M Shchuka, Lida Langroudi, Mariia Cherednychenko, Zoe E Gillespie, Andrew G Duncan, Ruxiao Tian, Natalia A Gajewska, Raphaël B Di Roberto, Jennifer A Mitchell

**Affiliations:** Department of Cell and Systems Biology, University of Toronto, Toronto, Ontario M5S 3G5, Canada; Department of Cell and Systems Biology, University of Toronto, Toronto, Ontario M5S 3G5, Canada; Department of Cell and Systems Biology, University of Toronto, Toronto, Ontario M5S 3G5, Canada; Department of Cell and Systems Biology, University of Toronto, Toronto, Ontario M5S 3G5, Canada; Department of Cell and Systems Biology, University of Toronto, Toronto, Ontario M5S 3G5, Canada; Department of Cell and Systems Biology, University of Toronto, Toronto, Ontario M5S 3G5, Canada; Department of Cell and Systems Biology, University of Toronto, Toronto, Ontario M5S 3G5, Canada; Department of Cell and Systems Biology, University of Toronto, Toronto, Ontario M5S 3G5, Canada; Department of Cell and Systems Biology, University of Toronto, Toronto, Ontario M5S 3G5, Canada; Department of Cell and Systems Biology, University of Toronto, Toronto, Ontario M5S 3G5, Canada; Department of Cell and Systems Biology, University of Toronto, Toronto, Ontario M5S 3G5, Canada

**Keywords:** enhancers, embryonic stem cells, CRISPR–Cas9, neural differentiation, transcriptomics, chromatin accessibility

## Abstract

Sex-determining region Y box 2 (Sox2) is a critical transcription factor for embryogenesis and neural stem and progenitor cell (NSPC) maintenance. While distal enhancers control *Sox2* in embryonic stem cells (ESCs), enhancers closer to the gene are implicated in *Sox2* transcriptional regulation in neural development. We hypothesize that a downstream enhancer cluster, termed *Sox2* regulatory regions 2–18 (SRR2–18), regulates *Sox2* transcription in neural stem cells and we investigate this in NSPCs derived from mouse ESCs. Using functional genomics and CRISPR–Cas9-mediated deletion analyses, we investigate the role of SRR2–18 in *Sox2* regulation during neural differentiation. Transcriptome analyses demonstrate that the loss of even 1 copy of SRR2–18 disrupts the region-specific identity of NSPCs, reducing the expression of genes associated with more anterior regions of the embryonic nervous system. Homozygous deletion of this *Sox2* neural enhancer cluster causes reduced SOX2 protein, less frequent interaction with transcriptional machinery, and leads to perturbed chromatin accessibility genome-wide further affecting the expression of neurodevelopmental and anterior–posterior regionalization genes. Furthermore, homozygous NSPC deletants exhibit self-renewal defects and impaired differentiation into cell types found in the brain. Altogether, our data define a *cis*-regulatory enhancer cluster controlling *Sox2* transcription in NSPCs and highlight the sensitivity of neural differentiation processes to decreased *Sox2* transcription, which causes differentiation into posterior neural fates, specifically the caudal neural tube. This study highlights the importance of precise *Sox2* regulation by SRR2–18 in neural differentiation.

## Introduction

Specialized cell types within the nervous system are generated during embryonic development from populations of self-renewing stem and progenitor cells that emerge from defined neurogenic zones (Johe *et al*. 1996; Molofsky *et al*. 2003). These neural stem and progenitor cells (NSPCs) migrate and undergo differentiation to create interconnected cellular systems consisting of neuronal and glial cell types, which include astrocytes and oligodendrocytes (Götz and Barde 2005; Shen *et al*. 2006). More than 80 different neuronal and glial cell types corresponding to developmental stage, regional specificity, and cellular function have been identified by single-cell gene expression patterns in the mouse postnatal brain and spinal cord (Rosenberg *et al*. 2018; Zeisel *et al*. 2018). To execute the complex functions of the vertebrate nervous system, regionally distinct gene expression profiles instruct NSPC populations to organize specialized cell types in the brain and spinal cord along the rostral–caudal and dorsal–ventral axes (Mathis and François Nicolas 2000; Hagey *et al*. 2016).

The proliferative activity, developmental potency, and region-specific identity of NSPCs are controlled by genetic programs defined by differential regulatory element usage (Ensini *et al*. 1998; Ziller *et al*. 2015). Cell fate decisions involve extensive transcriptional changes that are governed by gene regulatory networks—functional DNA sequences responsive to transcription factors (TFs), chromatin topology, and/or epigenetic modifiers (Brandenberger *et al*. 2004; Bailey *et al*. 2006; Loh *et al*. 2006). Enhancers are regulatory elements that influence gene expression in a spatiotemporal manner (Banerji *et al*. 1981). Enhancers can be positioned upstream or downstream and at variable genomic distances from the genes they regulate (Tuan *et al*. 1989; Sanyal *et al*. 2012). During cell differentiation, changes in TF binding occur at enhancer regions, which directly impact target gene expression and developmental outcomes (Wamstad *et al*. 2012; Kieffer-Kwon *et al*. 2013; Huang *et al*. 2016). Enhancers can regulate gene transcription through the formation of specific chromatinchromatin interactions (Carter *et al*. 2002; Tolhuis *et al*. 2002; Zhang *et al*. 2013). Distal enhancer elements can be targeted to promoters by architectural elements, either through the combined action of the zinc finger protein CCCTC-binding factor (CTCF) and the ring-shaped cohesin complex, or by CTCF-independent mechanisms (Tang *et al*. 2015; Weintraub *et al*. 2017; Taylor *et al*. 2022).

Enhancer sequence-containing regions exhibit a variety of functionally correlated chromatin states, which are often inferred from DNA methylation and posttranslational histone modifications (ENCODE Project Consortium 2012). Additionally, enhancers appear to be more dynamic than promoter elements across various cell types, tissues, and taxonomic groups (Heintzman *et al*. 2009; Roadmap Epigenomics Consortium *et al*. 2015; Villar *et al*. 2015). By leveraging our knowledge of epigenetically-informed chromatin states [e.g. Histone H3 lysine 27 acetylation (H3K27Ac)], TF occupancy, chromatin accessibility, and coactivator recruitment (e.g. Mediator complex, EP300) (Creyghton *et al*. 2010; Kagey *et al*. 2010; Rada-Iglesias *et al*. 2011) in the context of enhancers we can make predictions about enhancer activity. However, a single gene can be regulated by multiple enhancers active in different cellular contexts or by redundant enhancers with some degree of overlapping spatiotemporal function (Hay *et al*. 2016; Moorthy *et al*. 2017; Osterwalder *et al*. 2018; Taylor *et al*. 2022). As a result, identifying the locations of enhancers and determining their phenotypic role(s) is an ongoing challenge (reviewed in Tobias *et al*. 2021).

Sex-determining region Y box 2 (*Sox2*) encodes a TF that is initially expressed in the inner cell mass and epiblast of preimplantation embryos (Gubbay *et al*. 1990; Avilion *et al*. 2003). *Sox2* expression is downregulated upon pluripotent cell commitment toward endodermal or mesodermal lineages; however, its transcription is maintained within the neuroectodermal lineage, where SOX2 inhibits mesendoderm fate determinants (Ivanova *et al*. 2006; Kopp *et al*. 2008; Thomson *et al*. 2011). *Sox2* represents one of the SOXB1 family TFs (*Sox1–3*) which have partially overlapping DNA-binding activity in the neural ectoderm and the anterior gut endoderm (Taranova *et al*. 2006; Que *et al*. 2007; Cavallaro *et al*. 2008). The deletion of *Sox2* in the E10.5–12.5 forebrain produces viable mice with mild neurodevelopmental impairments at birth (Miyagi *et al*. 2008). Conversely, *Sox2* is essential for neurogenesis in the adult mouse hippocampal niche, which exhibits a Sonic hedgehog (Shh) signaling-dependent depletion of NSPCs in the absence of *Sox2* (Favaro *et al*. 2009). NSPCs display regional and temporal heterogeneity in vivo, with both quiescent and amplifying progenitor cell populations that retain self-renewal and differentiation capabilities when cultured in vitro (Reynolds and Weiss 1992; Garcia *et al*. 2004; Suh *et al*. 2007). Diffusible factors can induce NSPCs to shift positional neural cell identities along the rostral–caudal or ventral–dorsal axes; however, the primary allocation of embryonic cells to the anterior neuroepithelium which derives the brain and cervical spinal cord progenitors vs “posteriorized” neural progenitors that drive elongation of the neural tube is thought to occur in the vertebrate epiblast prior to neural induction (Bertacchi *et al*. 2013; Hallmann *et al*. 2016; Metzis *et al*. 2018). These forms of regionalized gene regulation in neural-fated progenitor populations are essential to prevent the specification of inappropriate or mixed cell identities.

*Sox2* and its genic flanking sequences fall within a synteny block on chromosome 3 (q26.33) in placental mammals (Zhang *et al*. 2022). Multiple regulatory elements within this conserved sequence block show regionally biased activities and are posited to coordinate *Sox2* transcription in diverse neurogenic contexts where SOX2 is required (Uchikawa *et al*. 2003; Uchikawa and Kondoh 2016). In mice, 2 enhancers located proximally to the *Sox2* transcriptional start site (TSS), known as SRR 1 and 2, have been shown to independently drive reporter gene expression in both embryonic stem cells (ESCs) and NSPCs (Zappone *et al*. 2000; Tomioka *et al*. 2002; Catena *et al*. 2004; Miyagi *et al*. 2004). Biallelic deletion of SRR1 in mouse embryos has been shown to induce a transient *Sox2* expression deficit in the anterior neural plate (Iwafuchi-Doi *et al*. 2011). *Sox2* haplodeficient mice with a monoallelic deletion of SRR1 on the intact *Sox2* allele display cerebral malformations and a decrease in Nestin (*Nes*)-positive hippocampal NSPCs (Ferri *et al*. 2004). Conversely, SRR2 is thought to recruit transcriptional repressors and decrease *Sox2* expression during in vitro differentiation of embryonic and neural stem cells (Li *et al*. 2012; Marqués-Torrejón *et al*. 2013). Another *Sox2* regulatory element homologous to the N1 enhancer in chickens is first activated in the caudal lateral epiblast of mouse embryos and later controls the fate of *Sox2*-expressing neuromesodermal progenitor (NMP) cells (Takemoto *et al*. 2006, 2011). However, there still exists a significant gap in understanding between the identification of *Sox2* regulatory elements and how the interplay between distinct *cis*-regulatory elements allows for the fine-tuning of transcription required for specific developmental fates.

The ESC-to-NSPC differentiation model provides a genetically tractable system for studying how epigenetic remodeling and chromatin organization influence neural gene regulatory networks (Phillips-Cremins *et al*. 2013). We have shown that the *Sox2* control region (SCR), a distal cluster of enhancers located at position +104–111 kb downstream of the *Sox2* TSS, regulates *Sox2* expression in mouse ESCs (Chen *et al*. 2012; Zhou *et al*. 2014). As pluripotent cells differentiate, TF binding is redistributed genome-wide and the chromatin state is altered at the SCR, including a loss of H3K27ac enrichment and long-range interactions with the *Sox2* promoter (pSox2) (Beagan *et al*. 2016; Thompson *et al*. 2022; Chakraborty *et al*. 2023). Leveraging the context-dependent activity of this model enhancer cluster, the *Sox2* locus has been extensively used in various studies that investigated long-range gene regulation in pluripotent stem cells (Brosh *et al*. 2023; Chakraborty *et al*. 2023; Platania *et al*. 2024). However, a more comprehensive understanding of how regulatory elements contribute to *Sox2* gene regulation in neural lineage commitment is still required. To determine *Sox2* regulatory mechanisms in neural cells, we investigated the contribution of candidate neural lineage *Sox2* enhancers using mouse ESC-derived NSPCs and CRISPR–Cas9-mediated genome engineering.

## Materials and methods

### Embryonic stem cell culture

F1 (*Mus musculus*/129 × *M. castaneus*) ESCs were obtained from Barbara Panning, University of California San Francisco (Mlynarczyk-Evans *et al*. 2006). Cells were maintained on 0.1% (mass/vol) porcine gelatin-coated plates and Dulbecco's modified Eagle medium (Thermo Fisher 11960044) supplemented with 15% (vol/vol) ESC-qualified fetal bovine serum (FBS) (Wisent Bioproducts 920040), 0.1 mM nonessential amino acids (Thermo Fisher 11140050), 1 mM sodium pyruvate (Thermo Fisher 11360070), 2 mM GlutaMAX (Thermo Fisher 35050061), 0.1 mM 2-mercaptoethanol (Thermo Fisher 21985023), 1X Penicillin-Streptomycin (Thermo Fisher 15140122), 1,000 U/mL recombinant leukemia inhibitory factor (LIF), 3 µM GSK3β inhibitor CHIR99021 (Millipore Sigma 361559-5MG), and 1 µM MEK inhibitor PD0325901 (Millipore Sigma 444966-5MG), referred to here as “LIF/2i medium”. Incubators were humidified and maintained at 37°C, 5% CO_2_ under normoxic conditions. All cultures were confirmed to be free of mycoplasma contamination during routine testing with the Mycoplasma PCR Detection Kit (BioVision Inc. K1476-100).

### *In vitro* neural differentiation and neural stem/progenitor cell culture

F1 mouse ESCs were differentiated toward NSPC in vitro according to established protocols (Ying *et al*. 2003). Briefly, ESCs were seeded into 10 cm gelatinized culture plates in LIF/2i medium (day 0) at a density of 1 × 10^4^ cells per cm^2^. On day 1, the culture medium was changed to a 1:1 mixture of Neurobasal medium (Thermo Fisher 21103049) and DMEM/F12 medium (Thermo Fisher 10565018) supplemented with 1X N2 (Thermo Fisher 17502048), 1X B27 without Vitamin A (Thermo Fisher 12587010), 2 mM GlutaMAX, 10 mM HEPES (Thermo Fisher 15630080), 0.015% Fraction V BSA (mass/vol), 0.1 mM 2-mercaptoethanol and 1X Penicillin-Streptomycin, referred to here as “N2B27 medium”. On day 5, adherent differentiating cells were detached with Accutase (Millipore Sigma A6964-500ML) and transferred to low adherence 10 cm plates in N2B27 medium supplemented with 10 ng/mL murine epidermal growth factor (EGF) (Peprotech Inc. 315-09-100UG) and 10 ng/mL fibroblast growth factor 2 (Cedarlane Labs CLCYT386), referred to here as “NSE medium”. On days 10–12, the cell aggregates formed in suspension culture were dissociated with Accutase and seeded onto T25 flasks coated with 100 µg/mL of poly-D-lysine (PDL) (Millipore Sigma 27964-99-4) and 10 µg/mL laminin (Millipore Sigma L2020) in NSE medium at a density of 2 × 10^4^ cells per cm^2^. This is considered passage 0 (p0) of neural stem/progenitor cell cultures. NSPCs were seeded into PDL- and laminin-coated T25 flasks in NSE medium at a density of 2 × 10^4^ cells per cm^2^ and passaged every 4–5 days for a maximum of 25 passages. NSPC multipotency was assessed by differentiation into neuronal, astrocyte, and oligodendrocyte lineages following removal of EGF from NSE medium for 1 day and later by culture in NeuroCult Differentiation Medium (Stem Cell Technologies 05704) for an additional 9 days.

### CRISPR–Cas9-mediated deletion and fluorescent reporter line generation

Cas9-mediated deletions were carried out as previously described (Moorthy and Mitchell 2016). Briefly, Cas9 targeting guide RNAs (gRNAs) flanking predicted enhancer regions are provided in Supplementary Table 1. gRNA plasmids were assembled in the gRNA Cloning Vector (a gift from George Church; Addgene plasmid # 41824), using the protocol described by Mali *et al*. (2013). The sequence of the resulting guide plasmid was confirmed by Sanger sequencing. For deletions, F1 ESC were transfected with 5 µg each of 5′ gRNA, 3′ gRNA, and pCas9_GFP plasmid (a gift from Kiran Musunuru; Addgene plasmid # 44719) (Ding *et al*. 2013). To tag *Sox2* at the C-terminal, we constructed a homology-directed repair donor template using Gibson assembly. The following sequences were assembled using the Gibson Assembly cloning kit (New England Biolabs E5510S): mCherry2-N1 amplicon (a gift from Michael Davidson; Addgene plasmid # 54517), P2A sequence through oligos, and homology arms amplified from F1 genomic DNA excluding the gRNA target site in the 3′ arm (Supplementary Table 2). For homology-directed repair knock-in, F1 ESC were transfected with 5 µg each of *Sox2* 3′ untranslated region (UTR) gRNA and pCas9_GFP plasmid, as well as 10 µg donor fragment purified from EcoRV restriction digest. Transfections were performed using the Neon Transfection System (Thermo Fisher Scientific). Forty-eight hours after transfection, cells were sorted on a BD FACSAria for GFP-positive cells (for deletion experiments) or mCherry-positive cells (for tag knock-in). The collected cells were seeded at 1 × 10^4^ per cm^2^ on 10 cm gelatinized culture dishes and grown for 5–6 days until large well-defined colonies formed. Isolated colonies were picked and propagated for genotyping and gene expression analysis. For DNA extraction from 96-well plates, cells were washed once with phosphate buffer saline (PBS) and subsequently lysed in a humidified chamber with 50 µL 1× cell lysis buffer using the prepGEM Universal Kit (PUN0100, MicroGEM), according to the manufacturer's recommendations. The 96-well plates were screened for deletions using allelic-specific qPCR-based assays. All deletions were confirmed by Sanger sequencing and analysis using primers 5′ and 3′ from the gRNA target sites. Single nucleotide polymorphisms (SNPs) within the amplified product confirmed the genotype of the deleted allele.

### Derivation of primary NSPCs from mouse embryonic brain tissue

All animal experiments were approved under animal use permit #20011749 by the University Animal Care Committee (UACC) of the University of Toronto. Embryo collections from wild-type CD-1 (CD1(ICR) from Charles Rivers) were made using timed mating. On E12.5, pregnant CD-1 females were euthanized by cervical dislocation, and embryos were harvested in prewarmed PBS containing calcium chloride and magnesium chloride (D1283 Millipore Sigma). Gross dissection of the embryonic brain rostral to the optic cup (excluding the hindbrain) was performed using a Leica stereomicroscope at 12.5× magnification. Tissue pieces were gently dissociated with Accutase and mechanical trituration with a P1000 micropipette. Enzymatic dissociation was stopped by dilution in the N2B27 base medium. Dissociated primary NSPCs in suspension were pelleted by centrifugation and resuspended in complete NSE growth medium for routine monolayer subculture on PDL- and laminin-coated T25 flasks.

### Immunofluorescence microscopy and proximity ligation assays

Cells grown on coated glass coverslips were fixed with 10% neutral formalin (Millipore Sigma HT501128) for 15 min. Cells were permeabilized and blocked with 10% FBS and 0.1% Triton-X100 in 1× PBS for 60 min at room temperature. Primary antibodies were applied for overnight incubation at 4°C at a dilution of 1:40. Cells were washed twice with 0.1% Tween-20 in PBS and secondary antibodies were applied at a dilution of 1:1,000 for 2 h. Primary and secondary antibodies were diluted in 0.2% FBS, and 0.15% Tween-20 in PBS. Antibody product identifiers and dilutions used for immunofluorescence are listed in Supplementary Table 3. Nuclei were counterstained with 5 ng/mL 4′,6-diamidino-2-phenylindole (DAPI) for 30 s. Following 5 washes in PBS, coverslips were mounted onto slides with Vectashield (Vector Laboratories H100010) and sealed using transparent nail polish. Epifluorescence imaging was performed on a Zeiss Axio Imager Z2 Upright Microscope (ZEISS), equipped with a X-cite Exacte light source (Excelitas Technologies) and a CoolCube 1 camera (MetaSystems). Raw images were processed in FIJI (Schindelin *et al*. 2012).

For in situ protein detection by proximity-dependent DNA ligation assays (Fredriksson *et al*. 2002), NSPCs were fixed and processed on coverslips as above. Primary antibodies against phospho-serine 5 carboxy-terminal domain (S5P CTD) RNA polymerase II (RNAPII) (Abcam ab5131; RRID: AB_449369) and SOX2 (R and D Systems MAB2018; RRID: AB_358009) (MAB2018, R&D Systems), were diluted 1:200 and 1:1,000 (respectively) into 0.2% FBS/0.1% Triton X-100 in PBS and incubated on coverslips overnight at 4°C. Following this, coverslips with NSPCs were washed 3 times in 0.1% Tween-20 in PBS for 5 min each. Duolink probes (mouse plus and rabbit minus) were prepared and applied as described by the manufacturer's instructions for the Duolink PLA kit Cy5 (Millipore Sigma DUO92013). Coverslips with NSPCs were mounted in VECTASHIELD Antifade Mounting Medium with DAPI. Images were collected using a Leica TCS SP8 confocal microscope and 100× magnification objective lens with oil immersion at constant exposure. Individual nuclei were identified using Imaris 7.1 (Oxford Instruments; RRID:SCR_007370). 3D models were rendered and proximity ligation amplification (PLA) focal signals were automatically counted per nucleus. All PLA experiments were carried out on at least 3 independent cultures of NSPCs and a minimum of 100 individual nuclei were imaged per genotype. Single antibody and PLA probe hybridization negative controls were also conducted.

### Intracellular flow cytometry

For intracellular antigen staining flow cytometry, cells were incubated with 1 µL of LIVE/DEAD Fixable Dead Cell Stain (Thermo Fisher L34963) per milliliter of culture media at 37°C for 30 min, followed by 2 washes with PBS. Cultures were dissociated using Accutase, collected, centrifuged at 400g for 4 min, and resuspended in >200 µL of 4% paraformaldehyde (PFA) for 10 min at room temperature. After washing out PFA with PBS, cells were resuspended in 500 µL 0.5% BSA in PBS. A permeabilization and staining buffer (0.5% BSA and 0.1% Triton X-100 in PBS) was prepared. Cells were counted, aliquoted at 0.5 × 10^6^ cells per tube and resuspended in permeabilization and staining buffer for 10 min. Directly conjugated antibodies targeting SOX2 (eFluor570) or an isotype control were added (Supplementary Table 3), and cells were incubated for 1.5 h at room temperature, protected from light. Cells were washed and resuspended in PBS + 0.5% BSA for data acquisition with a BD Fortessa analyzer. Population gating for single live cells and quantification of median SOX2 staining intensity was performed using FlowJo v10 (RRID:SCR_008520).

### RNA extraction and reverse-transcription quantitative PCR

Total RNA from cultured cells was isolated using an RNeasy RNA kit (Qiagen) and processed with TURBO DNA-free kit (Thermo Fisher AM1907). DNase-treated RNA was reverse transcribed with random primers using the high-capacity cDNA synthesis kit (Thermo Fisher 4368814). Target gene expression was monitored by quantitative PCR and normalized to *Gapdh* and *Eef2a* levels with gene-specific primers (oligonucleotide sequences provided in Supplementary Table 4). We monitored the expression of *Sox2* by allele-specific reverse-transcription quantitative real-time PCR (RT-qPCR) due to the presence of discriminatory SNPs within the *Sox2* transcript, as previously described (Zhou *et al*. 2014). For each biological replicate, qPCR reactions were performed in technical duplicate using SYBR Select Master mix (Thermo Fisher 4472908) and the CFX 384 Real-Time Detection system (BIO-RAD; RRID:SCR_018057). Expression levels were interpolated from a standard curve dilution series of F1 ESC genomic DNA using CFX Maestro Software. All RNA samples were confirmed not to have DNA contamination because no amplification was observed in reverse transcriptase-negative samples.

### Reporter plasmid construction

SRR2 and SRR107 were amplified from a Sox2 BAC (RP23-274P9) by PCR using Phusion polymerase (New England Biolabs E0553S) and cloned into the pJET1.2/blunt cloning vector (Thermo Fisher K1232) by Zhou *et al*. (2014). The remaining *Sox2* neural enhancer candidates were amplified from genomic DNA isolated from Bl6 mouse tissue (Jackson Labs) using the DNeasy Blood and Tissue Kit (Qiagen 69504). The 1.8 kb pSox2 was amplified from mouse genomic DNA by PCR using DreamTaq polymerase (Thermo Fisher EP0702) and ligated into the BanII site to replace the TATA-box minimal promoter of the pGL4.23 vector. All primers used in plasmid construction are provided in Supplementary Table 2. Enhancer sequences were amplified using primers containing 15 bp 5′overhangs homologous to the sequences flanking the NotI restriction site in the pGL4.23 luciferase reporter vector. Amplicons were cloned into the NotI site of the pGL4.23 vector with pSox2 by In-Fusion cloning (Takara Bio 639650). All plasmids were purified from bacterial culture using Presto Midi Plasmid Kit (Geneaid Biotech Inc. PIE025).

### Dual luciferase reporter assay

To evaluate enhancer activity in a luciferase reporter assay, F1 ESCs were seeded on gelatin-coated 96-well plates at a density of 10,000 cells per well. Alternatively, F1 NSPCs were seeded onto PDL/laminin-coated 96-well plates at a density of 50,000 cells per well. After 24 h, the cells were cotransfected using Lipofectamine 3000 (Thermo Fisher L3000008) with pSox2-pGL4.23 reporter vectors and pGL4.75 encoding Renilla luciferase (Promega E8411 and E6931) at a 50:1 molar ratio. The Renilla plasmid served as an internal control for transfection efficiency in each well. After 24 h, the spent growth medium was replaced with fresh growth medium, according to the cell-type transfected. Luciferase activity in cell lysates was assayed 48 h posttransfection using the Dual Luciferase Reporter Assay kit (Promega E1910), and the Fluoroskan Ascent FL microplate reader (Thermo Fisher Scientific). After background signal correction, the ratio of firefly/Renilla luciferase activity was calculated for each tested enhancer candidate and was normalized to that of the empty vector.

### Chromatin immunoprecipitation with sequencing retrieval and data processing

Raw H3K27ac chromatin immunoprecipitation with sequenci (ChIP-seq) data from embryonic mouse forebrain tissue samples at E14.5 were obtained from the ENCODE Project Consortium (2012) with data identifiers provided in Supplementary Table 5. Raw single-end ChIP-seq reads retrieved from the Sequence Read Archive (https://www.ncbi.nlm.nih.gov/sra) or the European Nucleotide Archive (https://www.ebi.ac.uk/ena/browser/home) are listed in Supplementary Table 6, which include data from several studies (Estarás *et al*. 2012; Lodato *et al*. 2013, 2014; Phillips-Cremins *et al*. 2013; Webb *et al*. 2013; Mateo *et al*. 2015; Sun *et al*. 2015; Bonev *et al*. 2017; Sessa *et al*. 2017; Braccioli *et al*. 2018; Bertolini *et al*. 2019; Quevedo *et al*. 2019; Pagin *et al*. 2021). ChIP-seq reads were assessed for quality, adapter content, and duplication rates with FastQC (Andrews 2010), trimmed with Fastp, and aligned to mm10 (Frankish *et al*. 2019) with Bowtie2 (Langmead and Salzberg 2012), with the following parameters: -N 1 --sensitive- --no-unal --threads 8 -x 700. Removal of duplicated reads and reads mapping to ENCODE mm10 blacklist regions were performed using Samtools (Li *et al*. 2009). For visualization purposes, we used deepTools bamCoverage (Ramírez *et al*. 2014) to generate coverage profiles normalized to a depth of 1 million reads. Peaks were called for each DNA-binding protein sample separately using MACS2 narrow peak-calling (Zhang *et al*. 2008).

### RNA-sequencing library preparation and data processings

Total RNA from cultured NSPCs was isolated and processed in the same way as samples prepared for reverse-transcription quantitative PCR assays. Total RNA was sent to The Centre for Applied Genomics (The Hospital for Sick Children, Toronto) for paired-end rRNA-depleted total RNA-sequencing (RNA-seq) (Illumina 2500, 125 bp). Raw RNA-seq reads from the public domain were retrieved from the GEO, which includes data from the following studies (Bertolini *et al*. 2019; Lattke *et al*. 2021). Raw reads were assessed for quality, adapter content, and duplication rates with FastQC and trimmed with Fastp v0.20.0 (Chen, Zhou, *et al.* 2018). Trimmed reads were aligned to mm10 (GENCODE M25) with STAR v2.9.0a (Dobin *et al*. 2013), with the following: parameters --alignEndsType Local-- --outFilterMultimapNmax 20. Exon-mapped reads were quantified using featureCounts (Liao *et al*. 2014) and imported into R v4.3.2 for differential expression analysis with DESeq2 (Love *et al*. 2014). Genes with an absolute log2 fold-change (FC) > 1 and false discovery rate-adjusted *P* < 0.05 in total exon-mapped read counts were considered differentially expressed. The “normalized counts” are derived from DESeq2's normalization method. This method calculates size factors for each sample to account for differences in sequencing depth. For visualization purposes, these normalized counts were transformed to Z-scores to facilitate comparison across genes with different expression levels.

For allele-specific RNA-seq, a hybrid version of mm10 was created with SNPsplit v0.3.4 (Krueger and Andrews 2016) using CAST/EiJ SNPs to N-mask discriminatory variants on a 129S1/SvImJ SNP-substituted reference based on GRCh38 from the Mouse Genome Project (dbSNP142). Trimmed reads were aligned to the hybrid N-masked assembly with STAR v2.9.0a, with the following parameters: --alignEndsType EndToEnd --outSAMattributes NH HI NM MD --outMultimapperOrder Random --outSAMmultNmax 1. Allele-specific sorting of alignment files was performed using SNPsplit with default parameters. Genes with an absolute log2FC > 0.5 and false discovery rate-adjusted *P* < 0.05 in allele-sorted read counts using DESeq2 were considered to have significant allelic imbalances.

The alignment files of all replicates per sample group were combined using Samtools merge and used to create bedgraphs that were normalized to counts per million (CPM) mapped reads using Deeptools bamCoverage (Ramírez *et al*. 2014).

### Assay for transposase accessible chromatin with sequencing library preparation and data processing

NSPC assay for transposase accessible chromatin with sequencing (ATAC-seq) multiplex library preparation and next-generation sequencing were performed at the Princess Margaret Genomics Centre, Toronto, Canada (www.pmgenomics.ca); 30,000–50,000 Sox2–mCherry^129^ and ΔSRR2–18^129/Cast^ NSPCs were dissociated with Accutase and washed once with 0.04% BSA in PBS. Intact nuclei were isolated and processed using standard ATAC-seq procedures developed by Buenrostro *et al*. (2013) with modifications to the lysis and transposition steps based on the OMNI-ATAC protocol by Corces *et al*. (2017). Demultiplexed raw ATAC-seq reads were processed using the ENCODE ATAC-seq pipeline (v.1.10.0) established by the Kundaje Lab (RRID:SCR_023100). In this pipeline, MACS2 was used for peak-calling, and conserved peaks in biological replicates were filtered using Irreproducible Discovery Rate (IDR; RRID:SCR_017237) (Li *et al*. 2011). Accessible chromatin regions identified by conservative thresholded IDR peaks were retained for downstream analyses. The alignment files of all replicates per sample group were combined using Samtools merge and used to create bedGraphs that were normalized to CPM mapped reads (CPM) using Deeptools bamCoverage. IDR peaks were imported into R and annotated to the nearest TSS of genes using the ChIPpeakanno package (Zhu *et al*. 2010). Normalization of read counts and analysis of differential accessibility for the union peak regions were performed using DiffBind package (Stark and Brown 2011). Peak intervals with *Q* ≤ 0.00001 and log2FC ≥ 1.5 were considered differentially accessible.

### Transcription factor motif enrichment

TF digital footprint analysis was performed using TOBIAS with standard settings (Bentsen *et al*. 2020) and a reference compendium of nonredundant mammalian TF motifs from the Jaspar 2022 release (Castro-Mondragon *et al*. 2022). Nonredundant motifs with an absolute log2FC > 0.2 and *Q* value < 0.01 were considered significantly enriched in each condition. Independent cell culture replicates (*n* = 3) were merged into a single BAM file for each treatment for Tn5 bias correction and footprint scoring. A consensus peak set was exported from DiffBind. ENCODE mm10 blacklist regions (ENCFF543DDX) were excluded (Amemiya *et al*. 2019). We used the BART (Binding Analysis for Regulation of Transcription) tool (Wang *et al*. 2018) to predict transcriptional regulators of the differentially expressed genes between parent NSPCs and ΔSRR2–18^129/CAST^ NSPCs. The analysis was performed using “bart2 geneset” command and the default parameters, and the top 100 predicted regulators were compared with the results from our ATAC-seq footprinting analysis.

### Gene set enrichment analyses

Gene set enrichment analysis on RNA-seq data was performed by ranking genes according to their log2FC and analyzed using the R package “clusterProfiler” (Yu *et al*. 2012). The function “enrichGO” was used with the following parameters: OrgDb = org.Mm.eg.db, keyType = ‘SYMBOL”, ont = ‘BP”, pAdjustMethod = ‘BH”, pvalueCutoff = 0.01, qvalueCutoff = 0.05. Gene Ontology (GO) analysis was performed on narrowPeak files of accessible genomic regions using the R package “chipenrich” (Welch *et al*. 2014) and the function polyenrich with the following parameters: genesets = GOBP, method = “polyenrich-weighted”, multiAssign = TRUE.

### Data visualization

Principle component analysis biplots were plotted using PCAtools R package (Blighe and Lun 2020). MA plots for allelic imbalances in gene expression were plotted using DESeq2 (Love *et al*. 2014). ATAC-seq signal enrichment in accessible chromatin was performed using NGSplot (Shen *et al*. 2014). Differential gene expression and differential motif scores were plotted using the EnhancedVolcano R package (Blighe *et al*. 2019). Correlation and clustering heatmaps were plotted using the pheatmap R package (Kolde 2012).

### Statistical analysis

Data were analyzed with R version 4.2.3 (R Core Team 2020) and the Tidyverse package (Wickham *et al*. 2019). Statistical methods, *P* values, or adjusted *P* values for each comparison are listed in the figure legend and/or in the corresponding results section. Assumptions of normality and homogeneity of variance were assessed with Shapiro–Wilk and Fligner–Killeen tests. For all experiments, sample size was determined empirically. Investigators were not blinded to allocation during experiments or outcome assessments.

## Results

### *Sox2* proximal regions are responsible for the enhancement of *Sox2* transcription during differentiation to neural stem/progenitor cells

**Fig. 1.**
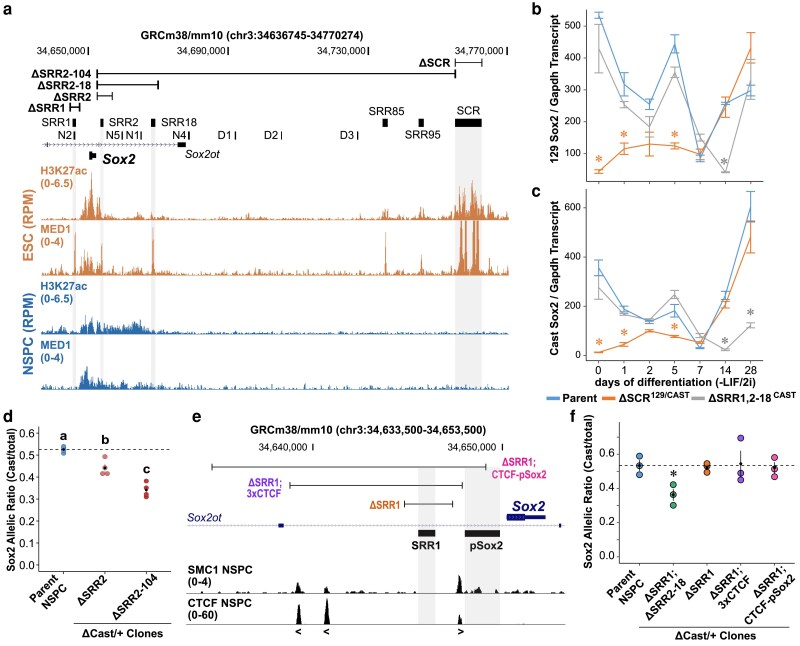
*Sox2* enhancer switch during mouse ESC neural differentiation. a) H3K27ac and MED1 ChIP-seq tracks in mouse embryonic stem cells (mESCs) and NSPCs visualized by UCSC Genome Browser (mm10). The *y*-axis represents signal intensity as RPM. Boxes aligned with shaded bars mark *Sox2* regulatory regions active in mESCs. Ranges show the margins of CRISPR–Cas9-mediated deletions of different SRRs or the SCR. b,c) *Sox2* expression in ESCs undergoing neural induction by allele-specific RT-qPCR comparing the isogenic F1 line (Parent) to lines harboring monoallelic (for SRR1,2–18) or biallelic (for SCR) deletions of enhancers with the indicated genotypes. Differentiation of naive mouse ESCs was induced by the removal of LIF and 2i from the culture medium (-LIF/2i) and the addition of FGF2 and EGF. Significantly different groups from the day 0 timepoint are indicated (∗) *P* < 0.05. Data represent mean ± SEM from independent cultures of isogenic parent line and mutant clonal cell lines; *n* ≥ 3. d) *Sox2* expression in stably derived NSPCs by allele-specific RT-qPCR comparing wild-type lines to lines harboring monoallelic deletions of enhancers with the indicated genotypes. Expression levels for each allele are shown relative to the total transcript levels. Groups determined to be significantly different (*P* < 0.05) from one another are labeled with different letters; to indicate *P* > 0.05, groups would be labeled with the same letter. e) NSPC SMC1 and CTCF ChIP-seq tracks visualized by the UCSC Genome Browser (mm10). Ranges show the margins of CRISPR–Cas9-mediated deletions of SRR1, clustered CTCF binding sites (3× CTCF), or the pSox2. Angled brackets indicate CTCF motif orientation. f) *Sox2* expression from the Cast allele in ESC-derived NSPCs by allele-specific RT-qPCR comparing the isogenic F1 line to lines harboring monoallelic deletions of enhancers with the indicated genotypes. Expression levels for each allele are shown relative to the total transcript level. Significant differences from the parent line values are denoted (∗) *P* < 0.05. Data represent mean ± SEM from independent cultures of isogenic parent line and mutant clonal lines; *n* = 3.

SRR1 and SRR2 exhibit enhancer activity in the mouse epiblast and in ESCs, suggesting their potential role in *Sox2* gene regulation early in development (Iwafuchi-Doi *et al*. 2011). Neither of these enhancers produce an obvious neurodevelopmental phenotype when both allelic copies are deleted in nonsensitized (*Sox2*^+/+^) genetically engineered mouse models (Ferri *et al*. 2004; Moncho-Amor *et al*. 2021). This raises the possibility that additional *Sox2* enhancer elements, including those already known to be conserved in amniotes that may be under selective pressure, could have partially overlapping or compensatory spatiotemporal activities that are not captured by conventional ectopic enhancer–reporter assays (Uchikawa *et al*. 2003). We aimed to identify *Sox2* enhancers active during early brain development. To do this, we reanalyzed ChIP-seq data from the ENCODE consortium. We focused on the coactivator-associated modification H3K27ac in 2 key contexts: mouse ESCs and E14.5 forebrain tissue (Fig. 1a; ENCODE dataset accession numbers provided in Supplementary Table 5) (ENCODE Project Consortium 2012). To refine our search to regions contributing to RNAPII-mediated transcription, we also reanalyzed Mediator subunit MED1 ChIP-seq data from in vitro generated NSPCs (Sequence Read Archive [SRA] and European Nucleotide Achive [ENA] dataset accession numbers provided in Supplementary Table 6) (Quevedo *et al*. 2019).

Examination of these ChIP-seq profiles showed that ESCs and NSPCs display distinct regions of H3K27ac enrichment surrounding the *Sox2* gene (Fig. 1a). The NSPC H3K27ac interval extends from the upstream flanking region, through SRR1, and into a 16.7 kb region downstream of the *Sox2* TSS containing SRR2 (we termed this downstream region SRR2–18) (Fig. 1a). MED1 chromatin recruitment signals were largely restricted to a 10 kb region immediately downstream of *Sox2* in cultured neural progenitors (Fig. 1a). We also observed H3K27ac enrichment in the SRR2–18 region in pooled E12.5 ganglionic eminence tissue from published forebrain epigenomic data (Rhodes *et al*. 2022) (Supplementary Fig. 1a), representing transient neural progenitor-rich niche regions in the ventral telencephalon that produce multiple neuronal subtypes in the cerebral cortex. These profiles of transcriptional coactivator activity suggested that *Sox2*-flanking regions, and SRR2–18 in particular, display enhancer-associated features in multiple neurogenic niches containing telencephalic NSPCs.

To investigate gene expression dynamics during neural differentiation, we modeled neural induction and NSPC generation using mouse hybrid F1 (*M. musculus*/129 × *M. castaneus*) ESC cultures. To validate this system, we used RT-qPCR to measure the levels of *Sox2*, *Pou5f1*, *Pax6*, and *Nes* RNA during differentiation to NSPCs (Supplementary Fig. 1b–e). Pluripotency marker *Pou5f1* transcript levels decreased significantly over the course of neural induction (*P* = 1.53 × 10^−2^, Wilcoxon test; Supplementary Fig. 1b), while neural lineage markers *Pax6* and *Nes* increased expression by day 5 of neural induction (*P* = 3.42 × 10^−5^ and 1.21 × 10^−5^, respectively, Welch's t-test; Supplementary Fig. 1c,d) (Lendahl *et al*. 1990; Suter *et al*. 2009). *Sox2* RNA levels peaked earlier at 3 days of neural induction (*P* = 2.47 × 10^−2^, Welch's t-test; Supplementary Fig. 1e) before stabilizing, consistent with its role in both pluripotent and neural progenitor states. Immunofluorescence imaging of monoclonal NSPC lines established from F1 ESCs confirmed the coexpression of both SOX2 and NES (Supplementary Fig. 1f).

We asked whether the combination of SRR1 and SRR2–18 plays a role in coordinating *Sox2* expression during neural lineage commitment. Previous CRISPR–Cas9-mediated deletion analyses by our group in mouse F1 ESCs established 2 different enhancer-deleted cell lines: one lacking both allelic copies of the SCR (ΔSCR^129/Cast^), and the other harboring monoallelic compound deletions of candidate enhancers located within 18 kb of the *Sox2* TSS (ΔSRR1,2–18^Cast^) (Zhou *et al*. 2014). The latter deletion encompasses most of the neural H3K27ac domain flanking the *Sox2* gene. To monitor *Sox2* expression from each allele and across a range of *Sox2* transcription levels, we used allele-specific RT-qPCR to determine the proportion of the sum *Sox2* transcript quantity detected from the 129 or Cast allele, as we have done previously (Moorthy *et al*. 2017). In line with our previous finding that reduced *Sox2* dosage in ΔSCR^129/Cast^ ESCs delays but does not abolish pan-neural progenitor gene activation during undirected differentiation (Zhou *et al*. 2014), *Sox2*-deficient ΔSCR^129/Cast^ cells showed an increase in *Sox2* expression during neural differentiation (*P* = 3.73 × 10^−2^, Wilcoxon test; Fig. 1b), and reached a level that was not significantly different from the unmodified control line at differentiation day 7 and in stable NSPC cultures (*P* = 0.46, Wilcoxon test; Fig. 1b). We did not observe any change in Cast *Sox2* transcript levels in ΔSRR1,2–18^Cast^ ESCs compared to the parent F1 ESCs and during the first 2 days of neural differentiation (*P* = 0.46 and *P* = 0.88, respectively, Wilcoxon tests; Fig. 1c). This is consistent with our previous finding that the *Sox2* proximal elements are dispensable for *Sox2* expression in pluripotent mouse ESCs (Zhou *et al*. 2014). Interestingly, ΔSRR1,2–18^Cast^ NSPCs showed decreased Cast-derived *Sox2* RNA compared to the unmodified, differentiation stage-matched controls (*P* = 2.86 × 10^−2^, Wilcoxon test; Fig. 1c). This temporal shift in enhancer dependency suggests that gene proximal enhancers become increasingly important for *Sox2* regulation as cells progress toward a neural fate.

We next asked whether any additional enhancer elements within the downstream flanking region function in combination with SRR2 to activate *Sox2* transcription in our NSPC model system. To this end, we generated clonal populations of ESCs harboring a large deletion of the ∼100 kb region spanning SRR2 and SRR104 (ΔSRR2–104^Cast^), a mutant generated to remove all the possible downstream enhancers between SRR2 and the SCR (Fig. 1a). We also established NSPCs from mutant ESCs with a monoallelic deletion of SRR2 alone (ΔSRR2^Cast^) and assessed allele-specific *Sox2* transcript levels. This allowed us to distinguish between the contribution of SRR2, an element with reported activity in epiblast stem cell-derived neural progenitors (Iwafuchi-Doi *et al*. 2011), and other known or novel elements within the SRR2–104 region (e.g. N5, N1, N4) (Fig. 1a). NSPC clones with either ΔSRR2^Cast^ or ΔSRR2–104^Cast^ genotypes showed a significant decrease in *Sox2* transcription from the targeted allele (*P* = 1.39 × 10^−2^ and *P* = 4.46 × 10^−4^, respectively, Tukey's test; Fig. 1d). Additionally, the proportion of Cast-derived *Sox2* transcript was further decreased in ΔSRR2–104^Cast^ cells compared to that in ΔSRR2^Cast^ NSPCs (*P* = 6.31 × 10^−3^, Tukey's test; Fig. 1d). Taken together, the differential effects on *Sox2* expression between ΔSRR2^Cast^ and ΔSRR2–104^Cast^ NSPCs suggests a degree of functional redundancy among enhancers within the SRR2–104 region downstream of *Sox2*.

The architectural domain harboring *Sox2* in pluripotent and neural cells is delimited on the centromeric end by 3 CTCF-bound regions upstream of the pSox2. We next focused on these upstream CTCF-bound sites that bookend SRR1, an enhancer in the anterior neural plate and in the dorsal telencephalon at mid-gestation (Zappone *et al*. 2000; Iwafuchi-Doi *et al*. 2011). To scrutinize the protein-binding elements with potential architectural and regulatory roles upstream of *Sox2*, we used ENCODE ChIP-seq data that profile CTCF binding in E14.5 mouse forebrain tissue (ENCODE Project Consortium 2012). We also reanalyzed publicly available ChIP-seq data for cohesin complex subunit structural maintenance of chromosomes 1 (SMC1) retention in purified bulk NSPCs (Phillips-Cremins *et al*. 2013). Each of the CTCF-bound regions upstream of *Sox2* showed SMC1 accumulation in NSPCs (Fig. 1e), raising the possibility that local chromatin topology contributes to *Sox2* expression in neural progenitors.

To test whether SRR1 and the promoter-proximal CTCF sites are required for *Sox2* expression in our ESC-derived NSPC model, we deleted these upstream regions encompassing SRR1 in F1 ESCs and differentiated genetically modified clones into NSPCs. As an external control, we confirmed the *cis*-linked decrease in *Sox2* allelic ratio in NSPCs established from ΔSRR1,2–18^Cast^ ESCs (*P* = 2.86 × 10^−2^, Welch's t-test; Fig. 1f). Deletion of SRR1 on the Cast allele (ΔSRR1^Cast^) did not significantly change the allelic ratio of *Sox2* transcripts compared to unmodified control NSPCs (*P* = 0.77, Welch's t-test; Fig. 1f). Moreover, we expanded the boundary of the SRR1-deleted region to include all 3 upstream CTCF binding sites in mouse neural cells (ΔSRR1–3xCTCF^Cast^). We did not observe any significant difference in *Sox2* allelic ratio in ΔSRR1–3xCTCF^Cast^ NSPCs (*P* = 0.90, Welch's t-test; Fig. 1f), which is consistent with our recent finding that disruption of pSox2 proximal CTCF sites in the E9.5–10.5 mouse embryo does not affect SOX2 levels in the central nervous system (CNS) (Chakraborty *et al*. 2023). It also remained a possibility that the extended CpG island promoter region contributes to strong baseline levels of *Sox2* expression in neural progenitors. Yet after establishing NSPC clones with a larger deletion (−15.6 kb to −1.1 kb of TSS) that spanned all proximal CTCF binding sites, SRR1 and greater than half of the CpG island promoter region on the Cast allele (ΔSRR1 CTCF-pSox2^Cast^), the fraction of *Sox2* transcript from the Cast allele did not significantly differ from that of control NSPCs (*P* = 0.79, Welch's t-test; Fig. 1f). In summary, our data suggest neither SRR1, the CTCF binding sites, nor the full-length CpG island promoter is required for *Sox2* transcription in this ESC model of neural lineage commitment.

### SRR2–18 is a *cis*-regulator of *Sox2* transcription and monoallelic deletion disrupts transcriptional regulatory programs in NSPCs

**Fig. 2.**
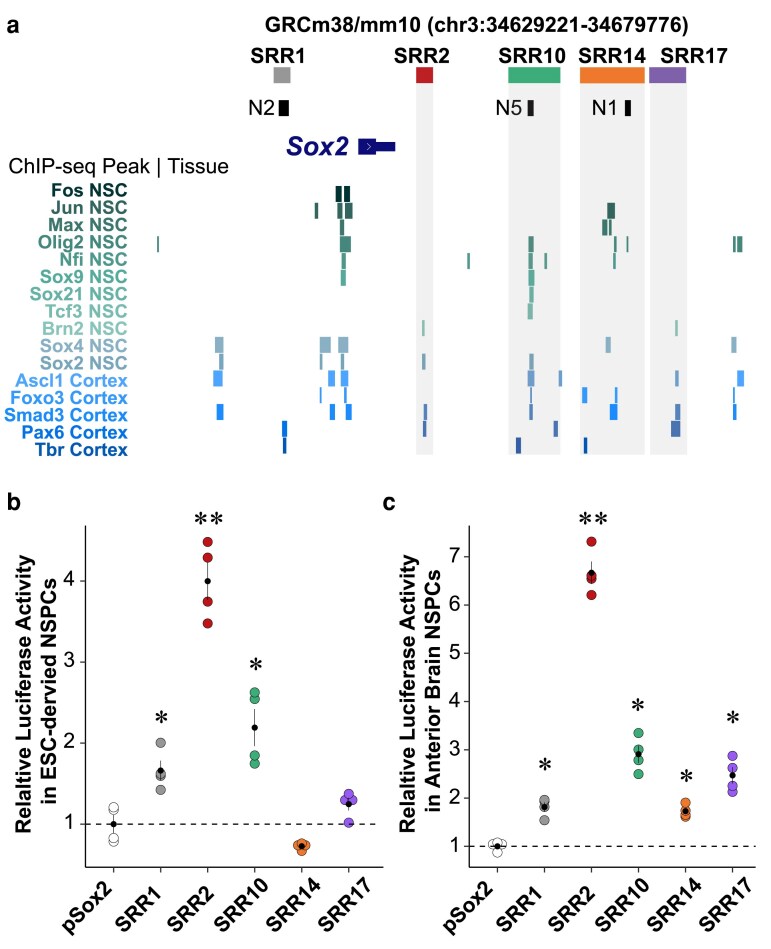
SRR2–18 contains multiple enhancers active in NSPCs from different sources. a) The SRR2–18 genomic region is displayed on the UCSC Genome Browser (mm10). SRRs (top) correspond to transcription factor-bound regions derived from NSPC and embryonic cerebral cortex ChIP-seq data sets compiled from public databases (bottom). b) Clusters of transcription factor binding sites in neural cells identifying distinct elements capable of activating transcription in luciferase reporter assays using F1 ESC-derived NSPCs. Dashed line indicates the mean activity of the pSox2 alone. c) The same luciferase reporter assays were performed in primary NSPCs generated from brain tissues dissected from mouse CD-1 embryos at E12.5. Data represent mean ± SEM from independent cultures of isogenic ESC-derived and primary NSPCs; *n* = 4. Significant differences from the empty pSox2 vector are indicated. (∗) *P* < 0.05, (∗∗) *P* < 0.01.

Since the H3K27ac- and MED1-enriched chromatin profiles in NSPCs were concentrated into a genomic window larger than a typical single active enhancer which is often predictive of spatially clustered enhancers (Chen *et al*. 2012), we next located the individual enhancers based on evidence of multiple TF binding in brain development using all publicly available TF ChIP-seq data in cultured neural progenitors or embryonic cerebral cortex tissue (SRA and ENA dataset accession numbers provided in Supplementary Table 6) (Estarás *et al*. 2012; Lodato *et al*. 2013, 2014; Webb *et al*. 2013; Mateo *et al*. 2015; Sun *et al*. 2015; Sessa *et al*. 2017; Braccioli *et al*. 2018; Bertolini *et al*. 2019; Pagin *et al*. 2021). Aside from the *Sox2* CpG island promoter, regions that were ∼+3.7 kb (SRR2), +9.8 kb (chick N5 homolog; hereafter SRR10), +14 kb (adjacent to the chick N1 homolog; hereafter SRR14), and +17.1 kb (hereafter SRR17) downstream of the *Sox2* TSS showed binding of TFs associated with neuroepithelial/radial glia identity (ASCL1, BRN2, PAX6, SOX2), stem cell homeostasis (FOXO3, JUN, MAX, SOX9, TCF3), and neurogenesis (FEZF2, NFI, OLIG2, SMAD3, SOX4, SOX21, TBR1) (Fig. 2a; coordinates provided in Supplementary Table 7). Notably, we observed only weak evidence of multiple TF binding at SRR1 (− 4.2 kb; chick N2 homolog) in these datasets.

We next performed luciferase reporter assays to assess the sufficiency of each enhancer candidate to induce transcriptional activation. To account for regulatory bias potentially attributable to native enhancer–promoter compatibility (Martinez-Ara *et al*. 2022), we modified the reporter vector to replace the minimal promoter with the mouse pSox2 (−2,209 to −347 bp of TSS) (Supplementary Fig. 2a). Control experiments carried out in ESCs or NSPCs with a set of previously validated enhancers, namely SRR107 (a submodule of the pluripotency-associated SCR) or the *Nes* intron 2 enhancer (Nes-I2) allowed us to verify that cell-type-selective enhancer activity is reproduced with an ectopic reporter approach (Josephson *et al*. 1998; Zhou *et al*. 2014) (Supplementary Fig. 2b). F1 NSPCs transfected with reporter plasmids carrying SRR1, SRR2, or SRR10 had significantly increased reporter activity compared to cells with the pSox2 plasmid alone (*P* = 6.11 × 10^−3^, 2.13 × 10^−4^, or 7.3 × 10^−3^, Welch's t-tests, Fig. 2b). To cross-validate the phenotype of F1 NSPCs in comparison to primary NSPCs established in the same EGF- and FGF2-containing culture medium, we generated NSPC lines from E12.5 whole brain tissue dissected rostral to the optic cup (including the telencephalon and the anterior diencephalon) from CD-1 mice. Despite the differences in genetic background, primary CD-1 NSPCs and F1 NSPCs show correlated (rho = 0.71, *P* = 3.04 × 10^−5^) activation of pSox2-driven reporter gene expression from regulatory regions found upstream (SRR1) and downstream (SRR2–18) (Fig. 2c). These results suggest that SRR1 is an active enhancer in NSPCs derived from F1 ESCs and embryonic forebrain/midbrain tissue from a reference mouse strain, but that *Sox2* transcriptional regulation could be distributed across multiple TF-bound active downstream enhancers.

**Fig. 3.**
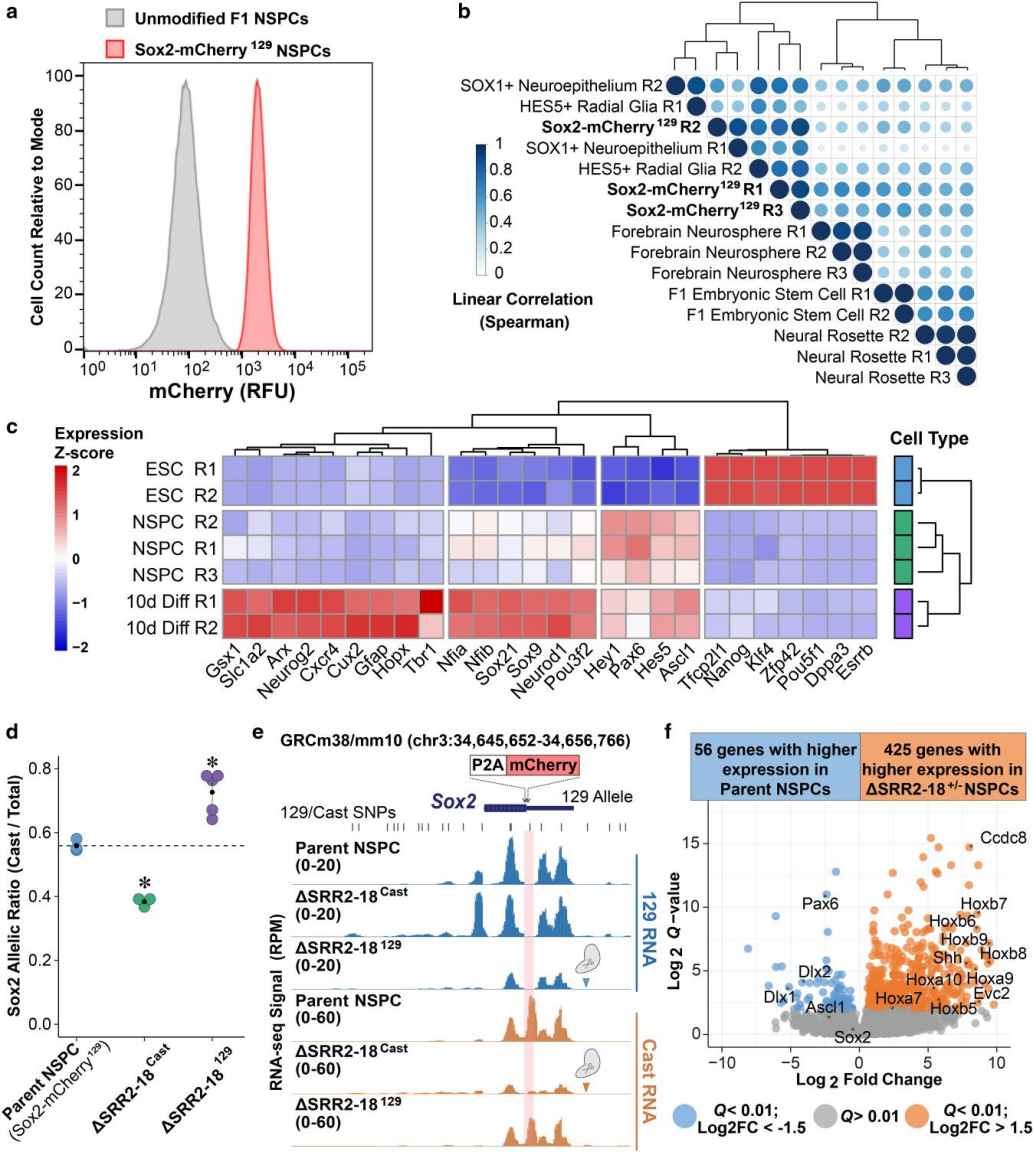
Monoallelic SRR2–18 deletion affects *Sox2* allelic dosage and positional identity of NSPCs. a) Distribution of mCherry fluorescence in *Sox2*–mCherry^129^ NSPCs overlayed with genetically unmodified NSPCs. b) Hierarchical clustering of sample correlations (Spearman's coefficient is represented by circle size and shade) based on normalized gene counts in bulk and transcription factor sorted subsets of neuroepithelial and neural progenitor cells from RNA-seq compiled from the GEO repository and this study. Each Sox2–mCherry^129^ isogenic replicate (R1–R3) was harvested at a different cell passage number. c) Hierarchical clustering of normalized gene counts across cell identity associated genes showing the patterns of gene expression changes between ESC-to-NSPC and NSPC-to-terminally differentiated cell (10d Diff) transitions. d) *Sox2* expression from the Cast allele in ESC-derived NSPCs by allele-specific RT-qPCR comparing the parent *Sox2*–mCherry^129^ lines to lines harboring monoallelic deletions of the SRR2–18 genomic region with the indicated genotypes. Expression levels for each allele are shown relative to the total transcript levels. Significant differences from the parent line values are indicated. (∗) *P* < 0.05. Data represent mean ± SEM from independent cultures of isogenic parent line and mutant clonal cell lines; *n* ≥ 3. e) Allele-sorted RNA-seq read pileup over the *Sox2* gene body, displayed on the UCSC Genome Browser (mm10). Shaded bar indicates discriminatory 129/Cast SNP on the 129 allele that is destroyed by the P2A-mCherry tag. Globular Cas9 protein scissor icon denotes allele harboring a deletion of the SRR2–18 genomic region. f) Volcano plot with the displayed number of differentially expressed genes between the parent and ΔSRR2–18^+/−^ NSPCs. Within the table, the number of genes that passed the cutoffs of absolute log2FC > 1.5 and *Q* < 0.01. *Q* values represent the adjusted *Q* values computed with the Benjamini and Hochberg method for controlling the false discovery rate (FDR). *P* values were extracted from a negative binomial regression model by Wald χ^2^ tests.

To test the role of this 16.7 kb downstream region independent of the upstream SRR1 region in neural lineage specification, we sought to decouple *Sox2* expression from SRR2–18 *cis*-regulation by CRISPR–Cas9-mediated deletion. We first generated an F1 reporter line with a mCherry insertion following the *Sox2* coding sequence and the cleavable P2A peptide on the *M. musculus*^129^ allele to facilitate the isolation of clonogenic NSPCs for extensive deletion analyses. Homogeneity of mCherry reporter gene expression (>97%) in NSPCs differentiated from the *Sox2*–mCherry^129^ parent line was confirmed by flow cytometry (Fig. 3a). Accordingly, we used total RNA-seq to analyze the transcriptome of the bulk *Sox2*-tagged NSPCs. We found high similarity in the transcriptome of *Sox2*–mCherry^129^ NSPC replicates across different passage numbers in culture (rho > 0.8 on raw counts and rho > 0.98 on log-transformed counts, Fig. 3b). We compared the *Sox2*–mCherry^129^ NSPCs with other types of mouse neural progenitor cells using available datasets and hierarchical clustering analysis. These *Sox2*-tagged NSPCs grouped with Sox1^+^ neuroepithelial cells and Hes5^+^ telencephalic radial glia, suggesting that *Sox2*–mCherry^129^ NSPCs maintain consistent gene expression profiles that are similar to other well-characterized neural progenitor cell types (Bonev *et al*. 2017; Baumann *et al*. 2019; Bertolini *et al*. 2019) (Fig. 3b). Terminal differentiations of these NSPCs were carried out to verify astrocyte, oligodendrocyte, and neuronal lineage specialization competence. We followed a 2-step protocol that involved cultivating *Sox2*–mCherry^129^ NSPCs and subclones in medium without exogenous EGF to facilitate exit from a self-renewing state with subsequent treatment with proprietary mixed lineage differentiation medium (NeuroCult Differentiation) for up to 10 days (Supplementary Fig. 3a). As anticipated, the progenies differentiated from the Sox2–mCherry^129^ NSPCs showed decreased expression of cell cycle process genes (*Mki67*, *E2f2*), consistent with cell cycle exit, and induced expression genes associated with astrocyte (*Aldoc*, *Gfap*, *Sox9*), oligodendrocyte (*Cxcr4*, *Nkx2-2*), and neuronal (*Rbfox3/NeuN*, *Slc1a2*, *Sox21*) maturation (Sandberg *et al*. 2005; Hatada *et al*. 2008; Patel *et al*. 2010) (Fig. 3c; Supplementary Fig. 3b and Table 8). These findings indicate that the mCherry-tagged NSPCs are capable of gene expression changes associated with differentiation into neuronal and glial cell types.

From the *Sox2*–mCherry^129^ parent line, we generated numerous ΔSRR2–18 heterozygous monoclonal cell lines (3 Cast and five 129 allelic deletions) to assess the *cis*-regulatory contribution of this enhancer cluster for *Sox2* expression and phenotype the deletion-harboring NSPCs. Allele-specific RT-qPCR analysis showed ΔSRR2–18^Cast^ NSPCs exhibited a significant decrease in the fraction of *Sox2* transcript from the Cast allele compared to NSPCs from the parent genetic background (*P* = 2.11 × 10^−4^, Welch's t-test; Fig. 3d). Reciprocally, NSPCs with a ΔSRR2–18^129^ genotype displayed a significant increase in the Cast *Sox2* allelic ratio compared to the control representing the parent genetic background (*P* = 5.50 × 10^−3^, Welch's t-test; Fig. 3d). These data indicate that the SRR2–18 region functions as a *cis*-regulator of *Sox2* in the neural lineage.

We next sought to investigate whether the monoallelic deletion of SRR2–18 specifically disturbs the allelic dosage of *Sox2* or if additional genes are also regulated in cis after this deletion. To account for mapping bias to reference mouse alleles, we assembled a SNP-substituted mouse genome using the 129S1/SvImJ substrain as a reference and N-masked discriminatory CAST/EiJ substrain SNPs (Supplementary Fig. 3c). We resolved the relative allelic expression at each locus to infer *cis* or *trans* modes of gene regulation. Profiles of allele-sorted RNA-seq reads showed that monoallelic deletion of SRR2–18 decreased steady-state *Sox2* transcription from the targeted allele (Fig. 3e). Differential allele-resolved expression analysis in monoallelic deletants identified *Sox2* as the only chromosome 3 gene regulated by the SRR2–18 cluster (Cast/Total ratio log2FC > |0.5|; *Q* < 0.05; Supplementary Fig. 3d). These data show that SRR2–18 only regulates *Sox2* expression in a *cis*-acting mechanism. This analysis also highlighted that imbalances in allelic dosage between the 129 and Cast haplotypes are pervasive across a wide range of gene expression levels in F1 NSPCs; however, these did not differ based on the targeted SRR2–18 allele.

Germline *Sox2* hypomorphism results in viable mice that present with posteromedial cerebral malformations and disrupted patterning of the anterior diencephalon (i.e. hypothalamus, pituitary) (Ferri *et al*. 2004; Kelberman *et al*. 2006; Langer *et al*. 2012). These models suggest that decreases in *Sox2* dosage below a certain threshold can negatively impact neural development. To test whether SRR2–18 mediated regulation of *Sox2* expression is required to establish the fundamental molecular properties of mouse NSPCs, we analyzed the total RNA-seq data in a manner agnostic to the hybrid cell haplotypes in ΔSRR2–18 heterozygous F1 NSPCs. Hereafter, analyses of pooled ΔSRR2–18^129^ and ΔSRR2–18^Cast^ sample data are referred to as ΔSRR2–18^+/−^. Differential expression analysis revealed reproducible alterations to the ΔSRR2–18^+/−^ NSPC transcriptome, which included 425 upregulated and 56 downregulated RNAs (log2FC > |1.5|; *Q* < 0.01) (Fig. 3f). ΔSRR2–18^+/−^ NSPCs showed increased expression of the *Hoxb* locus genes that are associated with caudal patterning of the neural tube (*Hoxb6–9*), ventral patterning of the neural tube (*Shh*, *Sim2*, *Evc2*), and transmembrane proteins involved in cell migration and morphogenesis (*Icam1*, *Dnah5*, *Tmem179*) (Fig. 3f; Supplementary Table 9). Parent Sox2–mCherry^129^ NSPCs had higher abundances of genes important for dorsal forebrain and midbrain development (*Pax6*, *Dlx2*, *Irx3*) (Fig. 3f) but also coexpressed genes associated with more caudal regions of the nervous system (*En2*, *Hoxa2*) (Supplementary Table 9). GO analysis revealed terms with top enrichment scores (ESs) among the upregulated gene set in ΔSRR2–18^+/−^ NSPCs, including, but not limited to, anterior/posterior pattern specification (ES = 0.69, *Q* = 3.1 × 10^−3^) and regionalization (ES = 0.61, *Q* = 7.5 × 10^−3^; Supplementary Fig. 4a and Table 10). Notably, ΔSRR2–18^+/−^ NSPCs show increased expression of *Wnt8a* which, along with *Wnt3a*, plays a niche-forming role in the development of caudal neural tissues (Ribes *et al*. 2009). In summary, NSPCs are sensitive to SRR2–18-driven *Sox2* transcriptional regulation, whereby ΔSRR2–18^+/−^ NSPCs activate caudal neural tube patterning pathways under steady-state culture conditions that normally maintain the self-renewal and identity of NSPCs from more rostral brain regions.

### Biallelic deletion of SRR2–18 disrupts NSPC self-renewal and multipotency

**Fig. 4.**
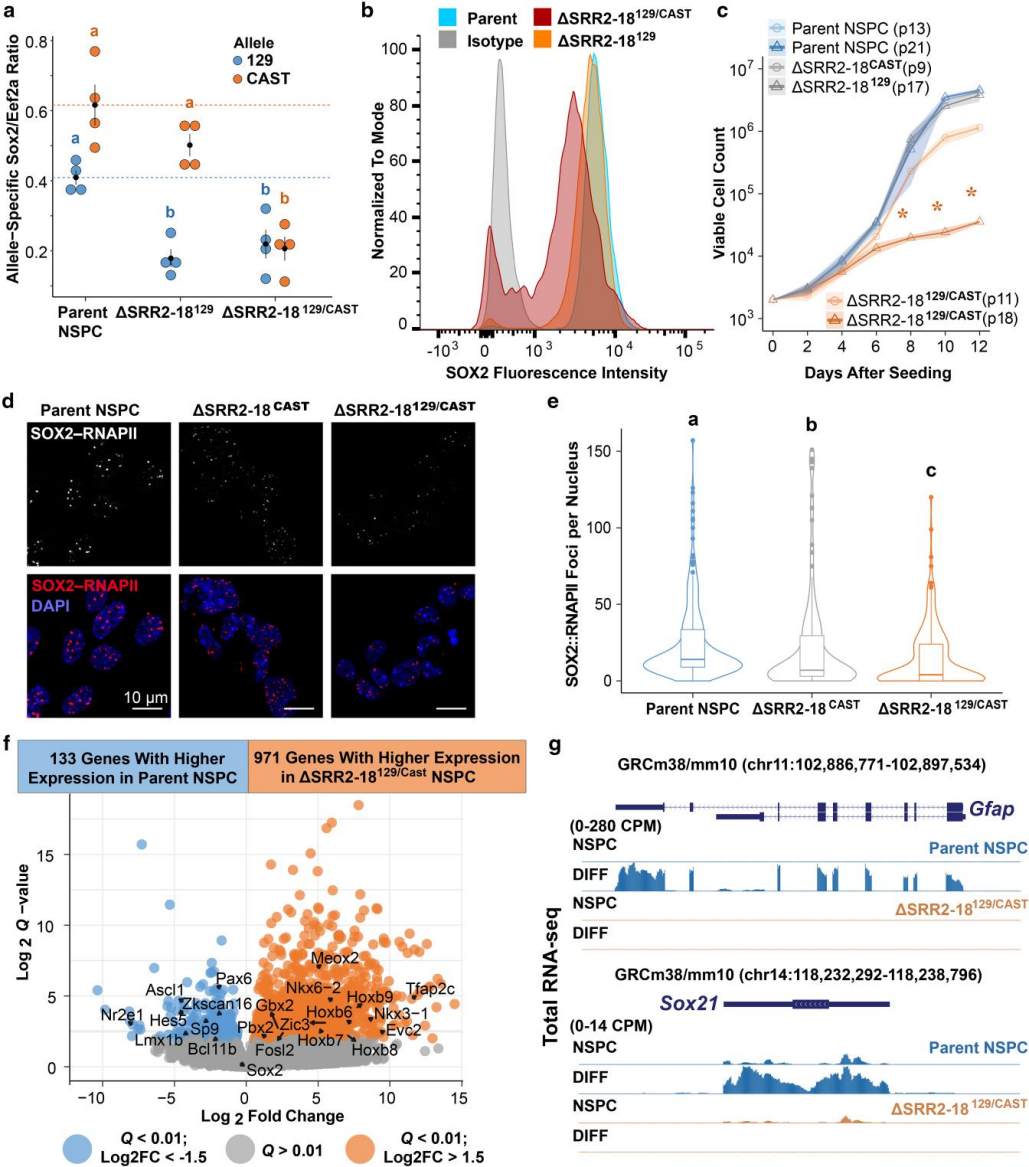
SRR2–18 is required for the maintenance of the NSPC phenotype and differentiation in vitro. a) *Sox2* expression in clonal NSPCs by allele-specific RT-qPCR comparing the indicated genotypes. Groups determined to be significantly different (*P* < 0.05) among multiple pairwise comparisons are labeled with different letters. To indicate *P* > 0.05, groups would be labeled with the same letter. Expression levels for each allele are shown relative to the reference transcript *Eef2a*. Data represent mean ± SEM from independent cultures of isogenic parent line and mutant clonal cell lines; *n* ≥ 4. b) Overlay histogram of flow cytometry data plotting the distribution of intracellular SOX2 level in the parent, ΔSRR2–18^129^, and ΔSRR2–18^129/Cast^ NSPCs. The filled gray curve represents NSPCs stained with the isotype control conjugated to the same fluorophore which acts as a negative control; *n* = 3. c) Cell proliferation curves from different clonal NSPC lines and passage numbers showing differences in acute growth observed in ΔSRR2–18^129/Cast^ NSPCs. Groups determined to be significantly different (*P* < 0.05) among pairwise comparisons to the parent line are labeled with an asterisk (*); *n* ≥ 4. d) PLA indicating the interaction between SOX2/RNAPII-S5P in the parent, ΔSRR2–18^129^, and ΔSRR2–18^129/Cast^ NSPCs. Images show median-intensity Z stack projections. Scale bar distance is 10 µm. e) Box-and-whisker plot related to (d) enumerating PLA foci per nucleus. Boxes indicate interquartile range of intensity values and whiskers indicate the 10th and 90th percentiles. Outliers are represented by dots. Images were collected from at least 3 biological replicate samples and over 100 nuclei were quantified for each denoted genotype. Groups determined to be significantly different (*P* < 0.05) from one another are labeled with different letters. f) Volcano plot with the displayed number of differentially expressed genes between the parent and ΔSRR2–18^129/Cast^ NSPCs that passed the cutoffs of absolute log2FC > 1 and *Q* < 0.01. g) Normalized RNA-seq tracks showing read pileup over the *Gfap* (astrocyte marker gene) and *Sox21* (neuronal maturation gene) loci, displayed on the UCSC Genome Browser (mm10). Signal tracks show greater transcript read coverage in libraries produced from the differentiated progeny (DIFF) of parent NSPCs to that of SRR2–18^129/Cast^ NSPC derivatives.

To investigate the phenotype of NSPCs established without the SRR2–18 enhancer cluster, we derived monoclonal ESC lines with a biallelic SRR2–18 deletion (ΔSRR2–18^129/Cast^) and differentiated them into NSPCs. ΔSRR2–18^129/Cast^ NSPCs maintain Nestin and SOX2 immuno-reactivity while displaying reduced *Sox2* transcripts produced from both alleles across multiple clones (*P* = 2.3 × 10^−2^ for 129 allelic mutants and *P* = 6.7 × 10^−3^ for Cast allelic mutants, Dunn's test with B–H adjustment, Fig. 4a, Supplementary Fig. 4b). Since the reduction in *Sox2* transcript level in ΔSRR2–18^129/Cast^ NSPCs was only 42% compared to parent NSPC clones (Fig. 4a), we next sought to determine if the distribution of SOX2 protein levels differed among parent, ΔSRR2–18^129^, and ΔSRR2–18^129/Cast^ NSPCs. Quantification of total intracellular SOX2 level by flow cytometry revealed that ΔSRR2–18^129/Cast^ NSPC cultures (40.0% decrease, *P* = 8.99 × 10^−7^, Welch's t-test), but not ΔSRR2–18^129^ heterozygous cells (10.2% decrease, *P* = 0.074, Welch's t-test), have significantly fewer SOX2-expressing cells compared to the control line with the same genetic background (Fig. 4b). Similarly, ΔSRR2–18^129/Cast^ NSPC populations show a pronounced decrease in the mean SOX2 abundance compared to the parent line (33.7% decrease, *P* = 4.89 × 10^−5^, Fig. 4b). Although SOX2 level tended to be lower in ΔSRR2–18^129^ NSPCs compared to the parent line, the effect was more subtle (15.0% decrease, *P* = 5.79 × 10^−2^, Fig. 4b). The observation that ΔSRR2–18^129^ heterozygous NSPCs have a comparable level of intracellular SOX2 to the parent line is surprising but may be explained by compensatory posttranscriptional or posttranslational mechanisms related to the caudal neural phenotype of these mutants at this developmental stage. To test whether the multimodal SOX2 distribution observed in ΔSRR2–18^129/Cast^ NSPCs contributes to the instability of the NSPC phenotype, we conducted growth curve analysis from multiple clones and different passage numbers. In maintenance culture conditions, ΔSRR2–18^129/Cast^ NSPCs initially show no difference in exponential-phase growth rate but adopt lower rates of cell division with prolonged time in culture (*P* < 7.8 × 10^−3^, Welch's t-tests, Fig. 4c), consistent with a self-renewal defect.

To obtain an accurate quantification of the functional relationship between SOX2 protein level and the frequency of transcriptional complex formation, we evaluated individual NSPCs by PLA with confocal microscopy (Dhaliwal *et al*. 2018, 2019). We quantified the protein interaction frequencies of SOX2 with RNAPII molecules engaged in the elongation of primary transcripts that are spatially resolved within single nuclei using an antibody targeting the phosphorylated serine 5 residue on the RNAPII CTD (Komarnitsky *et al*. 2000). In the parent NSPCs, SOX2 interacts with RNAPII at a median of 21 nucleoplasmic foci (Fig. 4d). We observed that SOX2–RNAPII interactions in ΔSRR2–18^Cast^ NSPCs were significantly decreased compared to NSPCs from the parent genetic background (median = 7, *P* = 6.55 × 10^−6^, Wilcoxon test; Fig. 4e). The frequency of SOX2–RNAPII association in NSPC nuclei were further decreased in the ΔSRR2–18^129/Cast^ line compared to that of monoallelic deletants (median = 4, *P* = 7.25 × 10^−3^, Wilcoxon test; Fig. 4e). Together, these data suggest that deletion of SRR2–18 affects the participation of SOX2 protein in transcriptionally productive complexes through reduced *Sox2* transcription.

We also expanded our RNA-seq transcriptomic analysis to include these NSPCs established from homozygous ΔSRR2–18^129/Cast^ ESCs, which revealed ΔSRR2–18^129/Cast^ cells were even more dissimilar to the parent control line in a principal component analysis of RNA-seq data than heterozygous ΔSRR2–18^−/+^ NSPCs (Supplementary Fig. 4c). To assess if the gene expression profiles of NSPCs decoupled from SRR2–18 mediated regulation are distinct from complete *Sox2* ablation, we compared our RNA-seq datasets to available transcriptome data from aggregates of self-renewing primary NSPCs (commonly referred to as neurospheres) established from wild-type and *Sox2*-ablated mice (Bertolini *et al*. 2019). Whereas *Sox2*^+/+^ neurospheres and our NSPCs from the *Sox2*–mCherry^129^ background grouped in a relatively small principal component space, the transcriptome of enhancer-deleted NSPCs markedly deviated from *Sox2*-knockout (KO) neurospheres (Supplementary Fig. 4c). Collectively, these findings suggest that regulatory perturbations of *Sox2* during neural differentiation lead to distinguishable transcriptomic signatures through the acquisition of alternative NSPC fates.

To identify transcript-level features that distinguish ΔSRR2–18^129/Cast^ NSPCs from the parent line, we performed differential gene expression analysis. A total of 133 transcripts showed a significant decrease in overall abundance and 791 transcripts showed a significant increase in biallelic SRR2–18 deletants (log2FC > |1.5|; *Q* < 0.01, Wald test under negative binomial model) (Fig. 4f; Supplementary Table 11). Annotation of the downregulated gene set in ΔSRR2–18^129/Cast^ NSPCs by GO analysis revealed enriched terms, including, but not limited to, nucleobase biosynthesis (ES = −0.41, *Q* = 2.8 × 10^−2^) and CNS neuron differentiation (ES = −0.52, *Q* = 3.0 × 10^−2^, 1-sided Fisher's tests) (Supplementary Fig. 4d and Table 12). Reciprocally, analysis of the induced gene set suggested that tube development (ES = 0.50, *Q* = 2.8 × 10^−3^) and positive regulation of MAPK cascade (ES = 0.57, *Q* = 1.3 × 10^−2^, 1-sided Fisher's tests) were among the top enriched biological processes in ΔSRR2–18^129/Cast^ NSPCs (Supplementary Fig. 4e). These data indicated that biallelic SRR2–18-deleted cells showed dysregulated nucleobase metabolism and MAPK pathways as well as altered neurodevelopmental signatures.

To assess whether biallelic deletion of SRR2–18 perturbs further differentiation into postmitotic CNS cell types, total RNA-seq was performed on bulk progenies differentiated from ΔSRR2–18^129/Cast^. Compared to differentiation of unmodified NSPCs, mixed lineage differentiation of ΔSRR2–18^129/Cast^ NSPCs displayed reduced expression of neuronal (*Sox21*, *Rbfox3*), astrocyte (*Gfap*, *Aldoc*), and early oligodendrocyte (*Cxcr4*) maturation-associated genes (Fig. 4g, Supplementary Fig. 5a). The differentiated progenies of SRR2–18^129/Cast^ cells exhibited pronounced differences in the number of differentially expressed genes (1,235 increased expression; 1,133 decreased expression; log2FC > |1.5|; *Q* < 0.01, Wald test under negative binomial model) compared to differentiation progenies of parent NSPCs (Supplementary Fig. 5b and Table 13), indicating a profound defect in gene regulation during differentiation. We observed a marked depletion of transcripts associated with biological processes involved in brain development, including, but not limited to, forebrain development (ES = −0.62, *Q* = 5.6 × 10^−5^, 1-sided Fisher's test), telencephalon development (ES = −0.60, *Q* = 2.8 × 10^−3^, 1-sided Fisher's test), and generation of neurons (ES = −0.31, *Q* = 7.4 × 10^−3^, 1-sided Fisher's test) in differentiated cells from a ΔSRR2–18^129/Cast^ background compared to those in differentiated cells with intact SRR2–18 (Supplementary Fig. 5c and Table 14). These results indicate that SRR2–18-mediated *Sox2* transcriptional regulation plays an important role in establishing a regional NSPC identity that supports differentiation into cell types found in the developing brain.

### Decoupling *Sox2* transcriptional regulation from SRR2–18 alters the accessible chromatin landscape of NSPCs

**Fig. 5.**
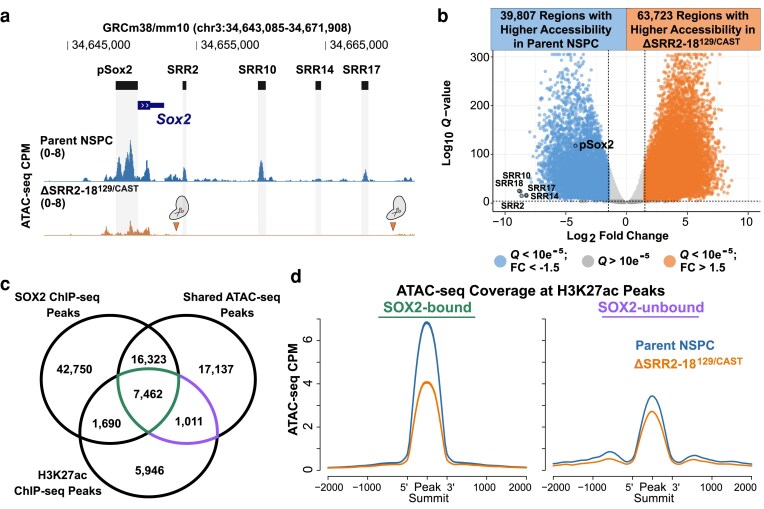
Deletion of SRR2–18 alters the accessible chromatin landscape of NSPCs. a) The *Sox2* gene and SRR2–18 genomic regions are displayed on the UCSC Genome Browser (mm10). pSox2 and SRRs (top) in NPSCs and ATAC-seq tracks from parent and SRR2–18^129/Cast^ NSPCs (bottom). Globular scissor protein icon denotes the margins of a biallelic SRR2–18 deletion. b) Volcano plot, with the number of differentially accessible genomic regions between the parent and ΔSRR2–18^129/Cast^ NSPCs. Within the table, the number of regions that passed the cutoffs of absolute log2FC > 1.5 and *Q* < 0.00001. *Q* values represent the adjusted *P* values computed with the Benjamini and Hochberg method for controlling FDR. *P* values extracted from a negative binomial regression model by Wald χ^2^ tests in DiffBind. c) Venn diagram showing the intersections between 3 peak sets: ATAC-seq data from this study, SOX2 ChIP-seq, and H3K27ac ChIP-seq. Raw ChIP-seq data were retrieved from the SRA database. For each dataset, peaks were called using MACS2, *Q* value cutoff of 0.05. Shared accessible chromatin regions between parent and ΔSRR2–18^129/Cast^ NSPCs were divided into SOX2-bound, SOX2-unbound, H3K27ac-modified, and non-H3K27ac-modified subsets. d) ATAC-seq peaks overlapping H3K27ac peaks were considered prospective *cis*-regulatory elements. Normalized ATAC-seq read coverage at SOX2-bound/H3K27ac and SOX2-unbound/H3K27ac accessible genomic regions between parent and ΔSRR2–18^129/Cast^ NSPCs.

SOX2 has a well-recognized role in supporting a permissive chromatin environment for the proper induction of neuronal lineage genes during neurogenesis (Amador-Arjona *et al*. 2015; Bertolini *et al*. 2019). We set out to obtain a comprehensive understanding of the gene regulatory network elicited downstream from *Sox2* decoupled from SRR2–18 regulation. We performed genome-scale identification and analysis of protein–DNA interaction sites inferred from accessible chromatin regions using ATAC-seq. Reproducible ATAC-seq peaks were largely distributed at intergenic regions, annotated TSSs, and gene bodies (Supplementary Fig. 6a,b). Consistent with their transcriptional dysregulation of *Sox2*, ΔSRR2–18^129/Cast^ NSPCs showed a decrease in chromatin accessibility at the pSox2 compared to that in parental cells (Fig. 5a). Analysis of ATAC-seq reads within the consensus peak set identified 88,910 regions with increased and 65,152 regions with decreased chromatin accessibility (log2FC > |1.5|; *Q* < 0.00001, Wald test under negative binomial model) (Supplementary Fig. 5b and Table 15). These data suggest that *Sox2* enhancer-perturbed NSPCs adopt a distinct regulome that reflects the unique spatial and functional identity of ΔSRR2–18^129/Cast^ cells.

We considered that those candidate regulatory elements represented in the set of decreased chromatin accessibility regions in ΔSRR2–18^129/Cast^ NSPCs were potential SOX2 targets in multipotent neural progenitors. To this end, we performed an intersection-based analysis using 3 datasets (Fig. 5c). We established a consensus set of ATAC-seq peaks shared between parent and ΔSRR2–18^129/Cast^ NSPCs, representing regions of accessible chromatin in both cellular contexts. We incorporated publicly available H3K27ac ChIP-seq peaks from Sox1–GFP^+^ in vitro differentiated NSPCs (Bonev *et al*. 2017), to focus on the subset of accessible chromatin regions enriched for active promoters and enhancers. Last, we reanalyzed NSPC SOX2 ChIP-seq data obtained from GEO to establish a nonredundant set of SOX2-occupied regions in NSPCs (74,233 unique regions; Supplementary Table 16). This allowed us to distinguish between regions of accessible chromatin associated with SOX2 regulation and loci with a similar chromatin profile but that are not direct SOX2 targets (Fig. 5c). The 3-way intersection of these datasets (7,462 retained peaks) represents genomic regions that are accessible, marked by H3K27ac, and bound by SOX2. Average profiles of ATAC-seq signal in the Sox2–mCherry^129^ NSPCs across qualifying genomic intervals showed enrichment of ATAC-seq signal at SOX2-bound regions [mean summit coverage = 6.7 reads per million (RPM)] compared to the signal at non-SOX2 target H3K27ac-modified regions (mean summit coverage = 3.2 RPM at 1,011 retained peaks) (Fig. 5d). As expected for an accessible and acetylated chromatin state, motif enrichment analysis of SOX2-bound regions predominantly recovered SOX, E26 transformation specific (ETS), and activator protein-1 (AP-1) motifs corresponding to the TF families previously recognized to contribute to NSPC self-renewal (Supplementary Table 17) (Pagin *et al*. 2021). Furthermore, SOX2-bound and H3K27ac-modified regions display a lower degree of ATAC-seq signal enrichment in ΔSRR2–18^129/Cast^ cells compared to that in parent NSPCs (Fig. 5d), which could reflect the reduction in SOX2 protein levels in these cells.

**Fig. 6.**
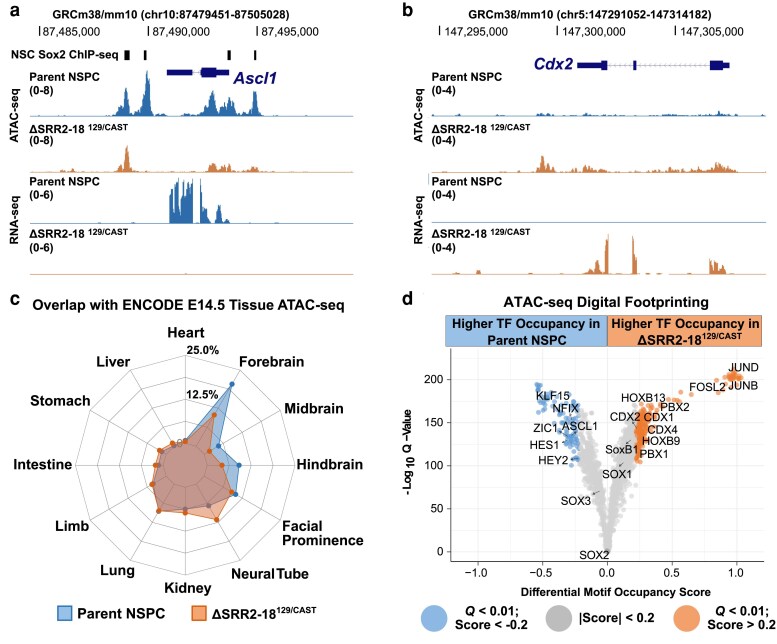
Induction of caudal patterning factors and chromatin remodeling in NSPCs decoupled from SRR2–18 mediated *Sox2* regulation. a) Normalized ATAC-seq and RNA-seq tracks over the *Ascl1/Mash1* gene and flanking genomic regions are displayed on the UCSC Genome Browser (mm10). Signal tracks show reduced accessibility at the *Ascl1* promoter and decreased total *Ascl1* RNA in ΔSRR2–18^129/Cast^ NSPCs. b) Normalized ATAC-seq and RNA-seq tracks over the *Cdx2* gene and flanking genomic regions displayed on the UCSC Genome Browser (mm10). Signal tracks show increased accessibility at the *Cdx2* locus and increased total *Cdx2* RNA in ΔSRR2–18^129/Cast^ NSPCs. c) Radar diagram of accessible genomic regions called from ATAC-seq data in parent NSPCs and SRR2–18^129/Cast^ NSPCs overlapping ENCODE consensus ATAC-seq peaks from E14.5 tissue samples. Height of the borders at each tissue intersection corresponds to the percentage of nonpromoter accessible genomic regions that overlap. d) Volcano plot, with the differential motif occupancy scores reflecting global changes in transcription factor binding in the consensus ATAC-seq peakset between the parent and ΔSRR2–18^129/Cast^ NSPCs from TOBIAS footprinting analysis. *Q* values represent the adjusted *P* values computed with the Benjamini and Hochberg method for controlling FDR.

To better understand the biological processes likely to be affected by altered protein–chromatin interactions genome-wide, GO analysis was performed on the differentially accessible regions annotated to the nearest TSS. Terms overrepresented in the set of ATAC-seq peaks showing decreased accessibility in ΔSRR2–18^129/Cast^ NSPCs were related, but not limited to, forebrain development (ES = −0.34, *Q* = 2.9 × 10^−10^, 1-sided Fisher's test) and neurogenesis (ES = −0.33, *Q* = 1.7 × 10^−35^, 1-sided Fisher's test) (Supplementary Fig. 6c and Table 18). Integrative analysis of the regulome and transcriptome in ΔSRR2–18^129/Cast^ NSPCs revealed that multiple proneural factors known to prime and regulate neuronal specification show a loss of transcriptional activation and chromatin accessibility, including Achaete-scute family TF 1 (*Ascl1*; also known as *Mash1*) (Fig. 6a). Reciprocally, genes near ATAC-seq peak regions with increased chromatin accessibility in ΔSRR2–18^129/Cast^ NSPCs included, but were not limited to, terms associated with regulation of anatomical structure morphogenesis (ES = 0.37, *Q* = 8.4 × 10^−38^, 1-sided Fisher's test), anterior/posterior pattern specification (ES = 0.31, *Q* = 3.0 × 10^−8^, 1-sided Fisher's test), and neural tube development (ES = 0.25, *Q* = 4.2 × 10^−4^, 1-sided Fisher's test) (Supplementary Fig. 6d and Table 19). We observed a specific increase in chromatin accessibility and transcription in *Hoxb* locus genes (*Hoxb5–9*) related to caudal neural tube patterning (Supplementary Fig. 6e), consistent with the regional identity bias of SRR2–18-deleted NSPCs observed by RNA-seq. In the developing vertebrate trunk, caudal-type homeobox factors function upstream of the *Hox* loci and are required for spinal cord specialization and patterning (Chawengsaksophak *et al*. 2004; Mazzoni *et al*. 2013). ΔSRR2–18^129/Cast^ NSPCs exhibit a switch-like gain of active transcriptional features at the *Cdx2* locus compared to the parent Sox2–mCherry^129^ line (Fig. 6b). These findings indicate that the induced expression of caudal positional factors and downregulation of factors involved in neuronal cell commitment observed in ΔSRR2–18^129/Cast^ NSPCs are supported by genome-wide alterations in the accessible chromatin landscape.

We explored the possibility that biallelic SRR2–18 deletion activates gene regulatory programs associated with posterior embryonic cell types. To assess the similarity of ΔSRR2–18^129/Cast^ NSPCs with tissue-specific chromatin accessibility signatures, we intersected our NSPC ATAC-seq peaks with a collection of embryonic day 14.5 (E14.5) excised mouse tissue ATAC-seq peaks from the ENCODE Consortium (Supplementary Table 5). *Sox2*–mCherry^129^ ESC-derived NSPCs show the greatest overlap in accessible chromatin intervals with forebrain ATAC-seq peaks (nonpromoter overlaps = 41,737, odds ratio = 114.8, *P* = 1.28 × 10^−35^, hypergeometric test; Fig. 6c), consistent with the extensive neurogenesis that occurs in the forebrain at this developmental stage. By contrast, accessible chromatin regions from ΔSRR2–18^129/Cast^ NSPCs were not significantly enriched in forebrain peaks (odds ratio = 1.7, *P* = 1, hypergeometric test; Fig. 6c) but did significantly overlap with the neural tube peak set (odds ratio = 2.9, *P* = 7.21 × 10^−11^, hypergeometric test; Fig. 6c). To further characterize the cell state acquired in ΔSRR2–18^129/Cast^ NSPCs, we performed hierarchical clustering on normalized ATAC-seq reads we reanalyzed from open access data on samples isolated from brain and spinal cord neural tissues (Supplementary Table 6) (Xu *et al*. 2018; Lattke *et al*. 2021; Delás *et al*. 2023). Although these bulk cell and tissue analyses fell short of pinpointing a specific developmental cell state associated with the accessible chromatin profile of ΔSRR2–18^129/Cast^ NSPCs (Supplementary Fig. 7a–c), the majority of ΔSRR2–18^129/Cast^ NSPC open chromatin regions form a partially overlapping cluster with moderately accessible genomic regions in ventral neural tube progenitors (cluster 7). GO terms from the nearest genes to cluster 7 regions are associated with chordate development and cell differentiation in the spinal cord (Supplementary Fig. 7a,b).

To assess how genome-wide protein–DNA interactions are altered in ΔSRR2–18^129/Cast^ NSPCs, we next identified the repertoire of TF motifs found at accessible chromatin regions. For this, we carried out digital footprinting, an approach that infers TF occupancy from ATAC-seq data based on the transposition frequency at base-pair resolution, after estimation and correction of positional bias (Bentsen *et al*. 2020). We found several dozen sequence-specific TFs with a differential occupancy score outside of statistical thresholds in NSPCs between the 2 genotypes (score > |0.2|, *Q* < 0.01; Fig. 6d, 2-sided 1-sample t-test under background distribution). Notably, SOX2 footprints were not differentially enriched between parental and ΔSRR2–18^129/Cast^ NSPCs which may be due to the remaining SOX2 protein in these cells; however, a relative lack of motif content differences across SoxB1 family members could not be ruled out. Moreover, functional compensation between coexpressed SoxB1 family TF in neural tissues is well documented (Graham *et al*. 2003; Miyagi *et al*. 2008). The motif footprints of several TFs that regulate NSPC proliferation and neuronal differentiation in various parts of the brain were markedly depleted in ΔSRR2–18^129/Cast^ NSPCs, including KLF15, NFIX, ASCL1, and the Notch downstream effectors HES1 and HEY2 (Fig. 6d). To assess the overlap between differentially expressed TFs and those with significantly different motif footprint scores, we used the set of differentially expressed genes from the parent NSPCs compared to the ΔSRR2–18^129/CAST^ NSPCs as a query set for BART (Binding Analysis for Regulation of Transcription) (Wang *et al*. 2018). We identified some of the same transcriptional regulators enriched in the ATAC-seq footprint analysis, specifically, ASCL1 and ZIC1::ZIC2 showed higher TF activity in unmodified parent NSPCs, while GRHL2 and SMAD3 displayed higher TF activity in ΔSRR2–18^129/CAST^ NSPCs (Supplementary Table 20). ATAC-seq footprinting analyses also highlighted several TF motifs that participate in the AP-1 complex and are enriched within NSPCs derived without SRR2–18 mediated *Sox2* regulation (Fig. 6d). Additionally, spinal cord specification and patterning factors such as CDX2/4, HOXB, and PBX1/2 also contribute to the altered chromatin accessibility profile in ΔSRR2–18^129/Cast^ NSPCs (Fig. 6d). These results indicate that decoupling *Sox2* expression from the SRR2–18 enhancer cluster induces a caudal region-specific identity. This phenotype is characteristic of cultured NSPCs driven by posterior regionalization factors in a regulatory network that is sensitive to *Sox2* transcript abundance and protein function.

## Discussion

The mechanisms of enhancer-mediated lineage- and cell-type-specific gene activation at different developmental stages or spatial contexts are unclear. Previous studies have suggested that enhancer switching is common during cell differentiation by comparing lineage-specific TF binding sites in stem/progenitor and mature cells (Soleimani *et al*. 2012; Alder *et al*. 2014; Chen, Liu, *et al*. 2018). We found that *Sox2*, a key factor in pluripotency and NSPC fate (Masui *et al*. 2007; Foshay and Gallicano 2008), exhibited a dynamic temporal pattern of *Sox2* RNA levels during mouse ESC to neural differentiation. Leveraging the genetic background of *M. musculus*/129 × *M. castaneus* ESCs and their derivatives, we demonstrated that these dynamics were coincident with a switch in regulatory control of *Sox2* from the pluripotency-associated SCR to SRR2–18, a proximal downstream region that determines *Sox2* transcription during the ESC-to-NSPC transition. Additionally, we found that the SRR2–18 region is necessary to maintain the appropriate level of *Sox2* transcriptional output in ESC-derived NSPCs under self-renewing conditions. Using an allelic deletion series, we showed that SRR2–18-mediated *Sox2* regulation is critical for sustaining the proliferative rate of NSPCs, establishing a more anterior region-specific neural progenitor identity, and activating neuronal differentiation genes. We identified patterns in differential chromatin accessibility and TF motif representation between parental and ΔSRR2–18^129/Cast^ NSPCs that propose how neural *Sox2* regulation safeguards a neurogenic program and a regionally biased neural progenitor identity through gene regulatory network activation.

Other investigations have described the regional selectivity of enhancer-mediated gene expression for candidate *Sox2* proximal enhancers in neural tissues using transgenic chick or mouse reporter assays (Uchikawa and Kondoh 2016). The proximal enhancers SRR1 and SRR2 show reporter gene activity in neurospheres and spatially restrict expression to the telencephalon in murine neural development (Zappone *et al*. 2000; Miyagi *et al*. 2004, 2006), however, neither SRR1 nor candidate distal enhancers (Beagan *et al*. 2017; Chakraborty *et al*. 2023) are sufficient to functionally compensate for the loss of SRR2–18-mediated regulation of *Sox2* in F1 NSPCs. To this point, we observed no difference in the level of *Sox2* knockdown in compound monoallelic ΔSRR1; ΔSRR2–18 NSPC clones (31–42%) and monoallelic ΔSRR2–18 NSPC clones (32–38%). A similar level of *Sox2* transcriptional deficiency in NSPCs of the developing forebrain has been described using a *Sox2* haplodeficient mouse model harboring a heterozygous deletion of SRR1 on the intact copy of *Sox2* (Ferri *et al*. 2004). However, in this case, reduced *Sox2* level in NSPCs in vivo was dependent on engineering a null *Sox2* allele. These findings indicate that, despite its functional sufficiency to activate gene transcription in primitive neural cell populations, SRR1 is dispensable for *Sox2* expression in a mouse NSPC model expressing *Sox2* biallelically.

Conditional biallelic deletion of the *Sox2* coding sequence resulted in impaired hippocampal NSPC survival and differentiation competence, as well as self-renewal defects in primary NSPCs upon extended maintenance in culture (Favaro *et al*. 2009; Bertolini *et al*. 2019). These studies highlighted the roles of immediate-early genes and cytokine signaling in the transcriptional amplification cascades linked to NSPC self-renewal. Decoupling *Sox2* regulation from the SRR2–18 enhancer cluster was permissive to neural induction; however, NSPCs harboring an SRR2–18 deletion on even 1 allele showed increased expression at multiple positional factor genes that specify the caudal identity of neural cells. The organizing stimulus of gene expression programs characteristic of caudal neural cell fates in adherent NSPCs is unclear; however, the increased expression of *Wnt8a* observed in mutant NSPCs could, if secreted into the culture microenvironment in a paracrine manner, promote the maintenance of caudally fated NSPCs. A reduction in *Sox2* dosage during axial regionalization in vivo has been reported to preconfigure the regulatory landscape of the caudal epiblast for WNT signaling input to specify posterior neural fates (Metzis *et al*. 2018; Blassberg *et al*. 2022). Additionally, fibroblast growth factor (FGF) signaling is involved in multiple stages of neural tube development, including specification of spinal cord identity, caudal extension and patterning, and directing of the onset of ventral neural tube identity and patterning (reviewed in Diez Del Corral and Morales 2017). However, given that all NSPC culturing media (containing exogenous FGF2 and EGF) are chemically defined and devoid of exogenous retinoids and Smoothened receptor ligands, the patterning responses observed in ΔSRR2–18^129/Cast^ NSPCs are consistent with cell-derived instructive factors. It will be interesting to examine if SRR2–18-mediated SOX2 regulation can acutely modify embryonic cell responses to FGF or WNT signaling during neural induction and axial regionalization.

Changes in chromatin accessibility or local genome topology can alter gene regulation programs during cell differentiation, leading to the establishment of unique spatial and functional cell identities (Thurman *et al*. 2012; Iwafuchi-Doi *et al*. 2016). SOX2 regulates the transcriptional permissiveness of its target genomic sites in multiple cellular contexts and facilitates regulatory chromatin–chromatin interactions in NSPCs that safeguard against depletion of the progenitor cell pool during brain development (Bertolini *et al*. 2019; Dodonova *et al*. 2020). In brain-derived neural progenitors, SOX2 transcriptionally primes a set of conserved basic helix–loop–helix TFs known as proneural genes, which induce neuron differentiation during brain development (Amador-Arjona *et al*. 2015). In this study, ESC-derived NSPCs exhibited transcriptome and accessible genome features similar to forebrain progenitors and showed primarily anterior brain tissue chromatin accessibility profiles. We observed specific patterns in genome-wide chromatin accessibility in ΔSRR2–18^129/Cast^ cells, which are associated with footprints for TFs that have previously established roles in the rostral–caudal regionalization of the nervous system. To our surprise, the highest scoring differential motif occupancy scores were classical immediate-early genes of the JUN, FOS, and ATF protein families. Components of the AP-1 complex, these multifunctional TFs play roles in cell proliferation, cell migration, neuronal maturation and activity, and the cell stress response (reviewed in Bejjani *et al*. 2019). Different genetically engineered mouse models deficient for stress- or mitogen-activated phosphorylases upstream of the AP-1 complex show defects in neural tube closure (Sabapathy *et al*. 1999; Chi *et al*. 2005); however, there are currently no high-confidence AP-1 TF family targets implicated in neural tube morphogenesis. SOX2 cooperates with distinct TF repertoires to target diverse genomic sites across different *Sox2*-expressing developmental fates, including in the spinal cord (Hagey *et al*. 2018). Given that ESC-derived NSPCs were propagated in adherent culture and FGF2-containing medium prior to transcriptome and regulome profiling, the temporal progression in regulatory mechanisms that direct this shift in target DNA recognition remains to be determined.

Our study focused on identifying the collective role of the SRR2–18 enhancer cluster in regulating *Sox2* during NSPC formation. This approach allowed us to capture the overall impact of this clustered regulatory region in our cellular model of neural induction, which may not be fully revealed by analyzing individual enhancers due to compensatory mechanisms. The function of the SRR2–18 cluster in maintaining *Sox2* expression in NSPCs likely contributes to the spatiotemporal control of *Sox2* levels during neural development. Future studies could employ combinatorial Cas9-mediated deletions or take a synthetic locus engineering approach to further dissect the contributions of individual enhancers within SRR2–18, while accounting for potential redundancy and compensatory effects. The disruption of region-specific NSPC identity observed after SRR2–18 deletion suggests that the action of SOX2 protein at target regulatory elements controlling anterior–posterior patterning genes, such as *Cdx2*, during differentiation is highly sensitive to *Sox2* transcriptional dosage. Furthermore, reduced *Sox2* levels cause a knock-on effect profoundly altering the transcriptional regulatory network and allowing the epigenome to adopt a more posterior neural phenotype. The SRR2–18 enhancer cluster appears to be a key node in this network, linking *Sox2* expression levels to the activation of region-specific genes. Our study thus provides a crucial piece in understanding how enhancer-mediated interactions contribute to the fine-tuning of gene expression necessary for proper neural development and regionalization.

## Supplementary Material

jkaf012_Supplementary_Data

## Data Availability

All cell lines and plasmids described are available upon request. The authors affirm that all data necessary for confirming the conclusions of this article are represented fully within the article and its tables and figures, supporting information, and the following data repositories. Raw sequencing data and processed bedGraph or narrowPeak files from this work were submitted to the GEO repository under the reference series GSE237778. All supplemental tables, along with the RT-qPCR, luciferase reporter, PLA immunofluorescence, and flow cytometry data for analysis and figure preparation, are available in the associated Zenodo repository (Tobias *et al*. 2025). This work does not use any original code. Supplemental material available at G3 online.
