# Supplementary material for: A Sox2 enhancer cluster regulates region-specific neural fates from mouse embryonic stem cells
Source: G3 (Bethesda). 2025 Jan 24;15(4):jkaf012. doi: 10.1093/g3journal/jkaf012 (PMC12005160; doi:10.1093/g3journal/jkaf012)
Supplement: jkaf012_Supplementary_Data [file jkaf012_supplementary_data.zip › Figure_S1_G3-2024-405518.pdf]

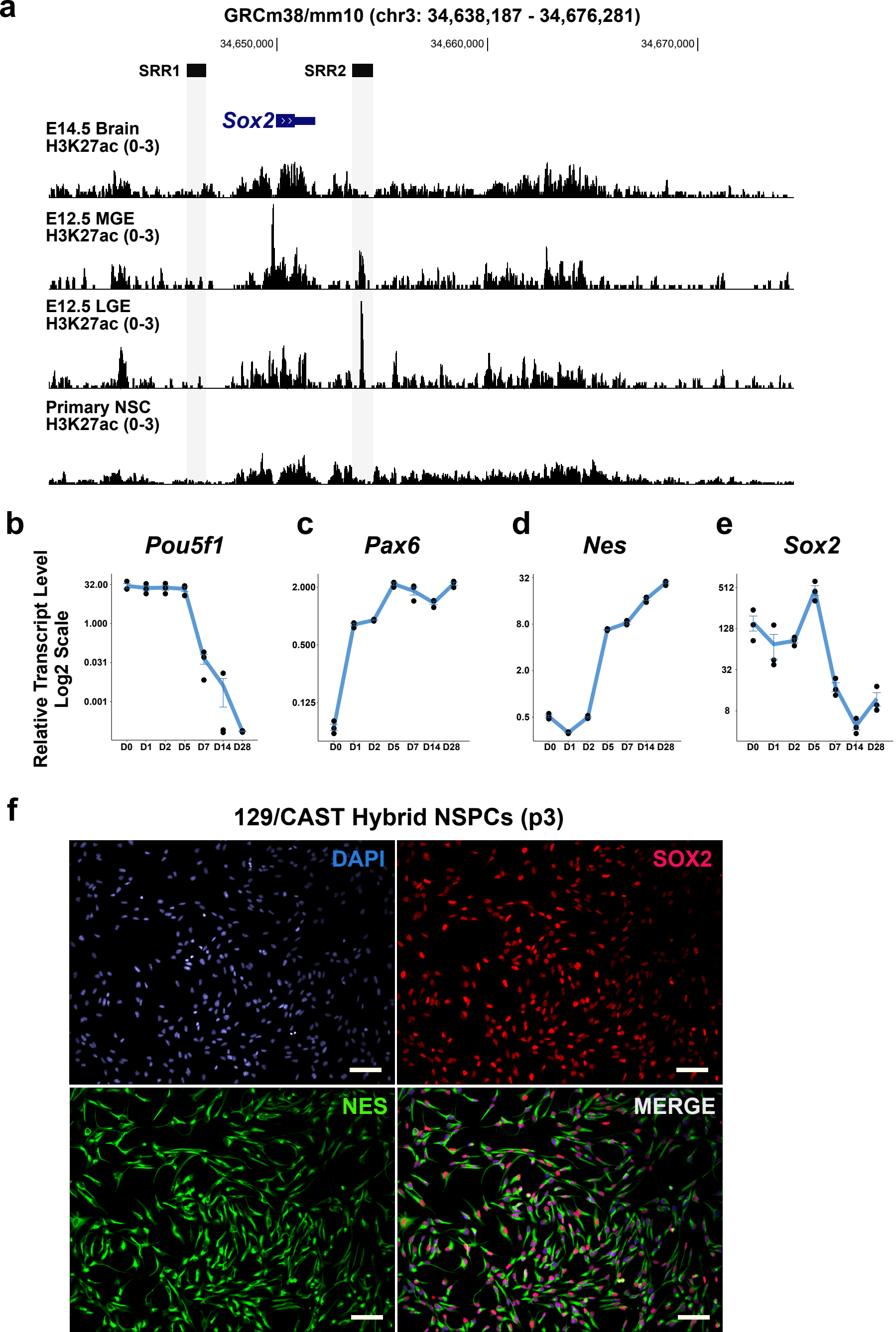
a
GRCm38/mm10 (chr3: 34,638,187 - 34,676,281)
34,650,000
34,660,000
34,670,000
SRR1
SRR2
Sox2
E14.5 Brain
H3K27ac (0-3)
E12.5 MGE
H3K27ac (0-3)
E12.5 LGE
H3K27ac (0-3)
Primary NSC
H3K27ac (0-3)
b
Pou5f1
Relative Transcript Level
Log2 Scale
32.00
1.000
0.031
0.001
D0 D1 D2 D5 D7 D14 D28
c
Pax6
2.000
0.500
0.125
D0 D1 D2 D5 D7 D14 D28
d
Nes
32
8.0
2.0
0.5
D0 D1 D2 D5 D7 D14 D28
e
Sox2
512
128
32
8
D0 D1 D2 D5 D7 D14 D28
f
129/CAST Hybrid NSPCs (p3)
DAPI
SOX2
NES
MERGE
