# Supplementary material for: A Sox2 enhancer cluster regulates region-specific neural fates from mouse embryonic stem cells
Source: G3 (Bethesda). 2025 Jan 24;15(4):jkaf012. doi: 10.1093/g3journal/jkaf012 (PMC12005160; doi:10.1093/g3journal/jkaf012)
Supplement: jkaf012_Supplementary_Data [file jkaf012_supplementary_data.zip › Figure_S4_G3-2024-405518.pdf]

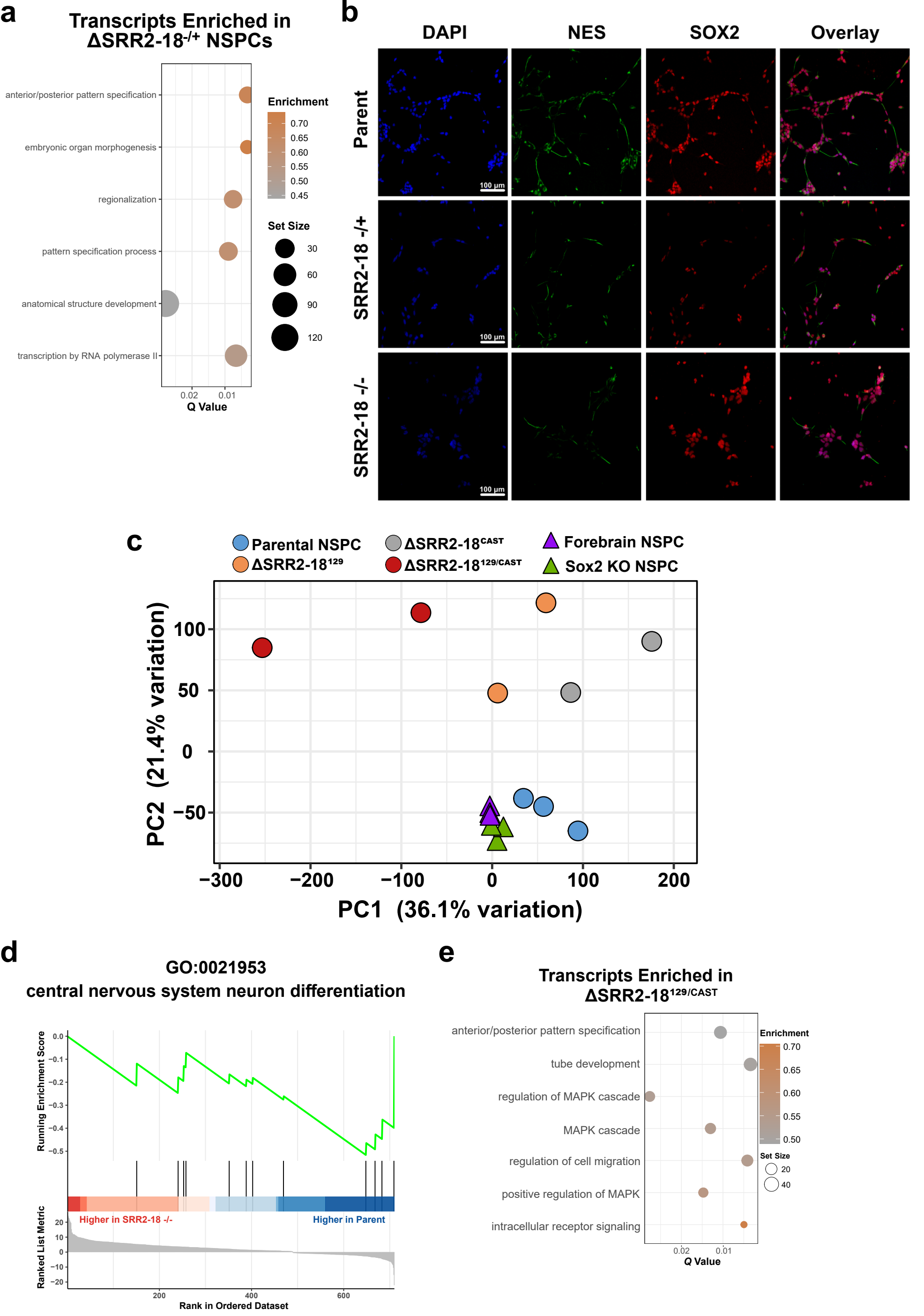

a
Transcripts Enriched in ΔSRR2-18-/+ NSPCs
anterior/posterior pattern specification
embryonic organ morphogenesis
regionalization
pattern specification process
anatomical structure development
transcription by RNA polymerase II
0.02
0.01
Q Value
Enrichment
0.70
0.65
0.60
0.55
0.50
0.45
Set Size
30
60
90
120
b
DAPI
NES
SOX2
Overlay
Parent
SRR2-18 -/+
SRR2-18 -/-
100 µm
c
Parental NSPC
ΔSRR2-18129
ΔSRR2-18CAST
ΔSRR2-18129/CAST
Forebrain NSPC
Sox2 KO NSPC
PC2 (21.4% variation)
PC1 (36.1% variation)
100
50
0
−50
−300
−200
−100
0
100
200
d
GO:0021953
central nervous system neuron differentiation
Running Enrichment Score
0.0
−0.1
−0.2
−0.3
−0.4
−0.5
Higher in SRR2-18 -/-
Higher in Parent
Ranked List Metric
20
10
0
−10
−20
200
400
600
Rank in Ordered Dataset
e
Transcripts Enriched in ΔSRR2-18129/CAST
anterior/posterior pattern specification
tube development
regulation of MAPK cascade
MAPK cascade
regulation of cell migration
positive regulation of MAPK
intracellular receptor signaling
0.02
0.01
Q Value
Enrichment
0.70
0.65
0.60
0.55
0.50
Set Size
20
40
