# Supplementary material for: A Sox2 enhancer cluster regulates region-specific neural fates from mouse embryonic stem cells
Source: G3 (Bethesda). 2025 Jan 24;15(4):jkaf012. doi: 10.1093/g3journal/jkaf012 (PMC12005160; doi:10.1093/g3journal/jkaf012)
Supplement: jkaf012_Supplementary_Data [file jkaf012_supplementary_data.zip › Figure_S6_G3-2024-405518.pdf]

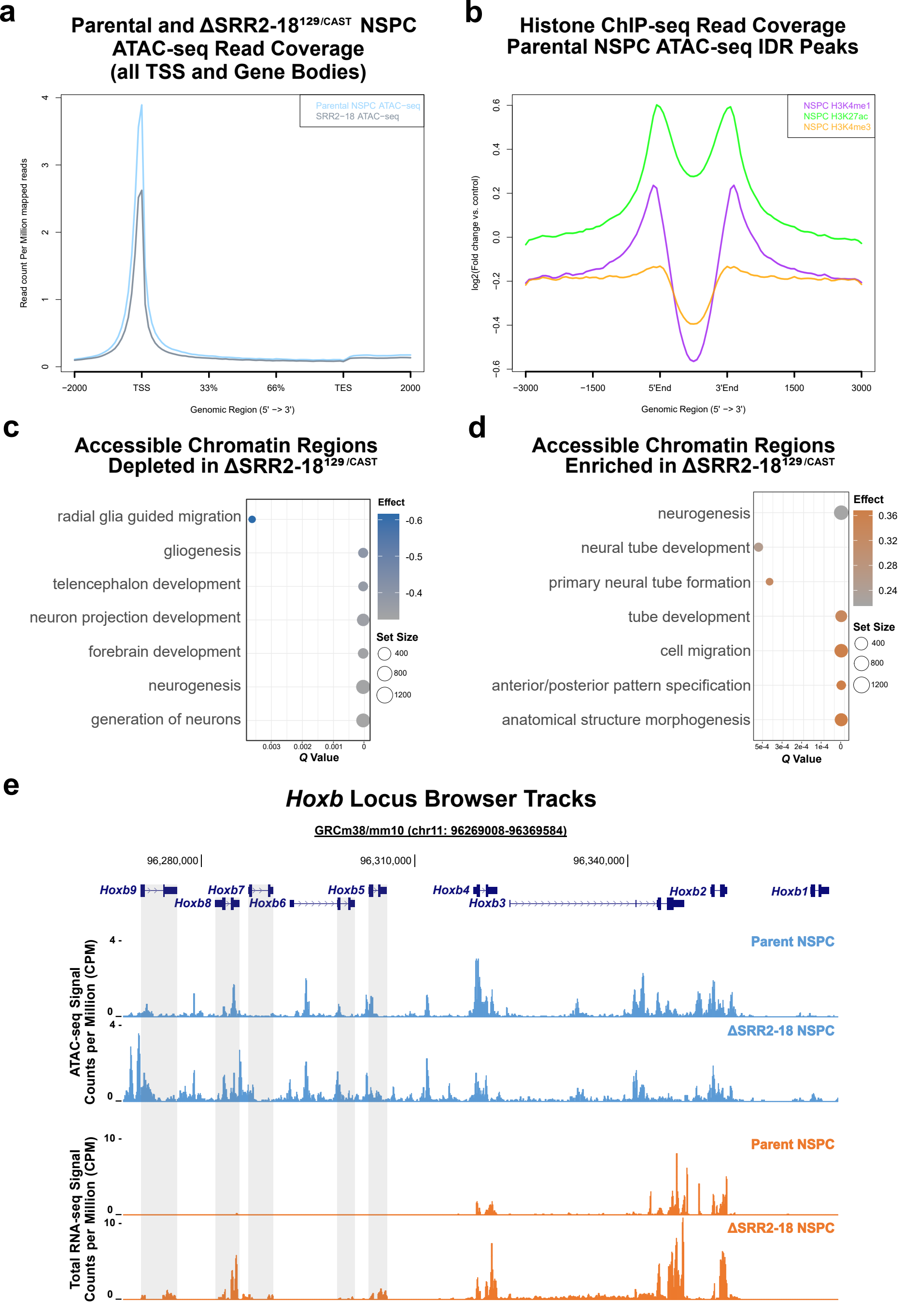
a
Parental and ΔSRR2-18129/CAST NSPC ATAC-seq Read Coverage (all TSS and Gene Bodies)
Parental NSPC ATAC−seq
SRR2−18 ATAC−seq
Read count Per Million mapped reads
−2000
TSS
33%
66%
TES
2000
Genomic Region (5' −> 3')
b
Histone ChIP-seq Read Coverage Parental NSPC ATAC-seq IDR Peaks
NSPC H3K4me1
NSPC H3K27ac
NSPC H3K4me3
log2(Fold change vs. control)
−3000
−1500
5'End
3'End
1500
3000
Genomic Region (5' −> 3')
c
Accessible Chromatin Regions Depleted in ΔSRR2-18129/CAST
radial glia guided migration
gliogenesis
telencephalon development
neuron projection development
forebrain development
neurogenesis
generation of neurons
Effect
Set Size
Q Value
d
Accessible Chromatin Regions Enriched in ΔSRR2-18129/CAST
neurogenesis
neural tube development
primary neural tube formation
tube development
cell migration
anterior/posterior pattern specification
anatomical structure morphogenesis
Effect
Set Size
Q Value
e
Hoxb Locus Browser Tracks
GRCm38/mm10 (chr11: 96269008-96369584)
96,280,000
96,310,000
96,340,000
Hoxb9
Hoxb8
Hoxb7
Hoxb6
Hoxb5
Hoxb4
Hoxb3
Hoxb2
Hoxb1
ATAC-seq Signal Counts per Million (CPM)
Parent NSPC
ΔSRR2-18 NSPC
Total RNA-seq Signal Counts per Million (CPM)
Parent NSPC
ΔSRR2-18 NSPC
