# Supplementary material for: A Sox2 enhancer cluster regulates region-specific neural fates from mouse embryonic stem cells
Source: G3 (Bethesda). 2025 Jan 24;15(4):jkaf012. doi: 10.1093/g3journal/jkaf012 (PMC12005160; doi:10.1093/g3journal/jkaf012)
Supplement: jkaf012_Supplementary_Data [file jkaf012_supplementary_data.zip › Figure_S7_G3-2024-405518.pdf]

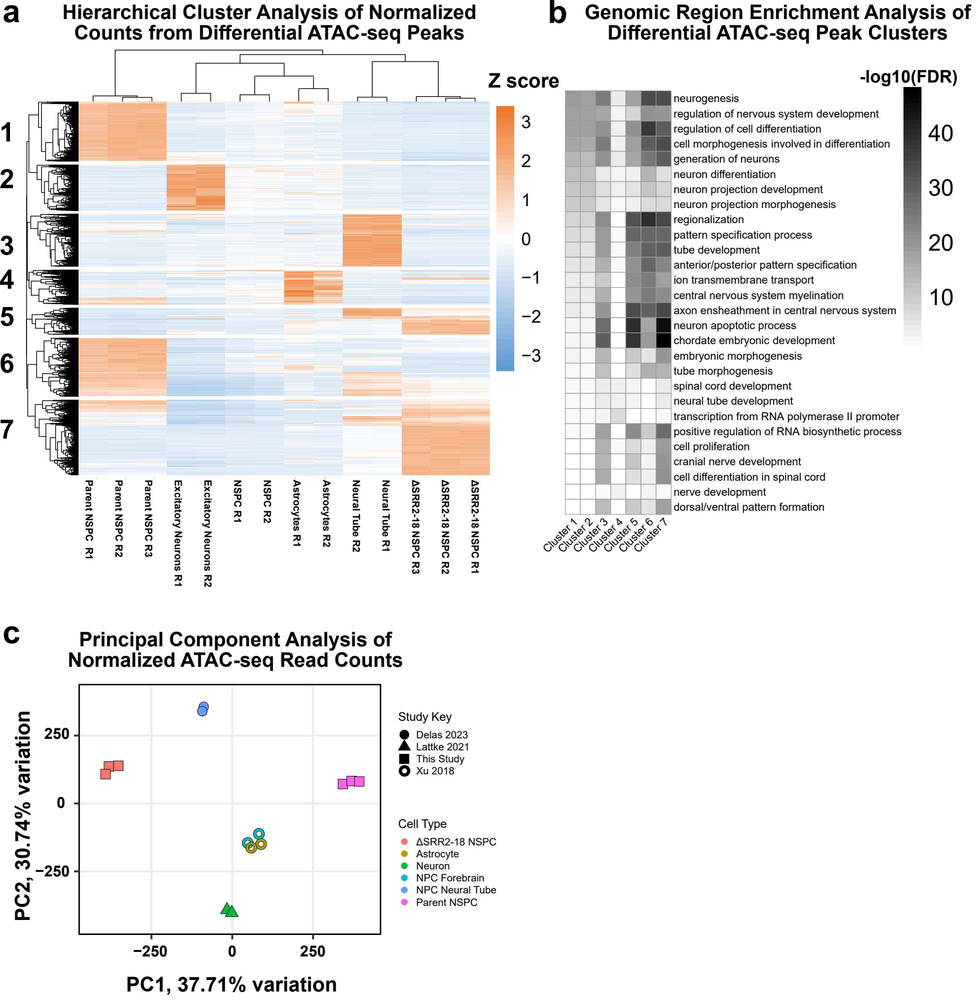
a
Hierarchical Cluster Analysis of Normalized Counts from Differential ATAC-seq Peaks
Z score
3
2
1
0
−1
−2
−3
1
2
3
4
5
6
7
Parent NSPC R1
Parent NSPC R2
Parent NSPC R3
Excitatory Neurons R1
Excitatory Neurons R2
NSPC R1
NSPC R2
Astrocytes R1
Astrocytes R2
Neural Tube R2
Neural Tube R1
ΔSRR2-18 NSPC R3
ΔSRR2-18 NSPC R2
ΔSRR2-18 NSPC R1
b
Genomic Region Enrichment Analysis of Differential ATAC-seq Peak Clusters
-log10(FDR)
40
30
20
10
neurogenesis
regulation of nervous system development
regulation of cell differentiation
cell morphogenesis involved in differentiation
generation of neurons
neuron differentiation
neuron projection development
neuron projection morphogenesis
regionalization
pattern specification process
tube development
anterior/posterior pattern specification
ion transmembrane transport
central nervous system myelination
axon ensheathment in central nervous system
neuron apoptotic process
chordate embryonic development
embryonic morphogenesis
tube morphogenesis
spinal cord development
neural tube development
transcription from RNA polymerase II promoter
positive regulation of RNA biosynthetic process
cell proliferation
cranial nerve development
cell differentiation in spinal cord
nerve development
dorsal/ventral pattern formation
Cluster 1
Cluster 2
Cluster 3
Cluster 4
Cluster 5
Cluster 6
Cluster 7
c
Principal Component Analysis of Normalized ATAC-seq Read Counts
PC2, 30.74% variation
250
0
−250
PC1, 37.71% variation
−250
0
250
Study Key
Delas 2023
Lattke 2021
This Study
Xu 2018
Cell Type
ΔSRR2-18 NSPC
Astrocyte
Neuron
NPC Forebrain
NPC Neural Tube
Parent NSPC
