# Supplementary material for: A Sox2 enhancer cluster regulates region-specific neural fates from mouse embryonic stem cells
Source: G3 (Bethesda). 2025 Jan 24;15(4):jkaf012. doi: 10.1093/g3journal/jkaf012 (PMC12005160; doi:10.1093/g3journal/jkaf012)
Supplement: jkaf012_Supplementary_Data [file jkaf012_supplementary_data.zip › Supplemental_Material_Legends_G3-2024-405518.docx]

**Supplemental Material Legends**

**Supplemental Figures**

**Figure S1. *Sox2* flanking region H3K27ac ChIP-seq profiles and neural lineage marker RT-qPCR.** a) H3K27ac ChIP-seq tracks visualized by UCSC Genome Browser (mm10). Shaded bars mark known *Sox2* regulatory regions (SRRs) active in mouse embryonic stem cells. Regions of H3K27ac enrichment located further upstream (-8 kb) and downstream of *Sox2* (+10 to +16 kb) are enhancer candidates in the whole brain (E14.5), the E12.5 medial (MGE) and lateral (LGE) ganglionic eminences in cortical neurogenesis, and in isolated neural stem/progenitor cells (NSPCs). b) *Pou5f1*, c) *Pax6*, d) *Nes*, and e) *Sox2* expression during F1 hybrid ESC differentiation to NSPCs by RT-qPCR. Differentiation of naïve mouse ESCs was induced by the removal of leukemia inhibitory factor and 2i from the culture medium and the addition of FGF2 and EGF. Expression levels are shown relative to internal control transcripts *Gapdh* and *Eef2a*. Data represent mean ± standard error of the mean (SEM) from independent cultures of isogenic parent line. n ≥ 3. (f) Immunofluorescence of microscopy of NES overlayed with SOX2 in F1 NSPCs. Scale bar distance is 50 µm.

**Figure S2. *Sox2* enhancer activity in NSPCs and ESCs in luciferase reporter assays with the *Sox2* promoter.** a) Luciferase reporter activity values following F1 NSPC transfection with the original minimal promoter (pMin) or modified with the *Sox2* promoter (pSox2) by restriction cloning. b) Fold increase in luciferase reporter activity in F1 ESCs and F1 NSPCs with enhancer insertion at the NotI restriction site. Dashed line represents empty pSox2 vector luciferase activity values. Enhancers are *Sox2* regulatory region 107 (SRR107), Nestin intron 2 enhancer, and SRR2. Data represent mean ± SEM from independent ESC or NSPC cultures of isogenic F1 line. n ≥ 3. (∗) P < 0.05, (∗∗) P < 0.01, (∗∗∗) P < 0.001, (∗∗∗∗) P < 0.0001, (ns) not significant.

**Figure S3. Morphological and genomic analysis of *Sox2*-mCherry tagged NSPCs and their differentiated progeny.** a) Representative morphologies in cultured cells by phase contrast microscopy. F1 hybrid Sox2-mCherry^129^ tagged neural stem/progenitor cells (NPSC) are shown in maintenance culture conditions and differentiation medium (NeuroCult™ medium with differentiation supplement). b) Volcano plot and the number of differentially expressed genes between the F1 NSPCs and the progeny of the same cells after 10 days of undirected differentiation. The colored data points show the transcripts passed the cutoffs of absolute log2 fold-change > 1 and Q < 0.01. c) Schematic of computational approach to sort next-generation sequencing alignment data overlapping single nucleotide polymorphisms between the 129 and Cast sub-strains N-masked in the custom hybrid genome assembly. d) Plots of log2 fold changes versus the average expression signal (MA-plots) showing significant allelic imbalances in allele-sorted RNA-seq data comparing ΔSRR2-18^129^ versus parent NSPCs and ΔSRR2-18^Cast^ versus parent NSPCs. Known and predicted genes on chromosome 3 are labeled.

**Figure S4. Characterization of transcriptomic perturbation in SRR2-18 deleted in NSPCs.** a) Bubble plot of the enriched GO biological process terms of genes nearest to the chromatin regions showing increased accessibility in SRR2-18^129/Cast^ vs. parent NSPCs. b) Qualitative SOX2 and NES immunofluorescence micrographs of parent NSPCs, ΔSRR2-18 heterozygous, and ΔSRR2-18 homozygous NSPCs. Scale bar is 100 µm. c) Biplot of principle component analysis comparing RNA-seq data of parent and enhancer-deleted NSPCs to wild-type and Sox2 knockout (KO) neurosphere RNA-seq data compiled from the GEO repository d) Enrichment plot showing depletion of genes annotated by gene ontology identifier GO:0021953 (central nervous system neuron differentiation) in ΔSRR2-18^129/Cast^ vs. parent NSPCs. e) Bubble plot of the significantly enriched gene sets of upregulated genes in ∆SRR2-18^129/Cast^ vs. parent NSPCs.

**Figure S5. Neural lineage markers changes upon SRR2-18 deletion in differentiating NSPCs.** a) Normalized RNA-seq tracks over the *Aldoc* (astrocyte marker gene), *Rbfox3/NeuN* (neuronal marker gene), and *Cxcr4* (oligodendrocyte marker gene) loci, displayed on the UCSC Genome Browser (mm10). Signal tracks show greater transcript read coverage in libraries (in counts per million [CPM]) produced from the differentiated progeny of parent NSPCs to that of ΔSRR2-18^129/Cast^ NSPC derivatives. b) Volcano plot and the number of differentially expressed genes between the differentiated progeny of parent versus ΔSRR2-18^129/Cast^ cells. Colored data points show the transcripts passed the cutoffs of absolute log2 fold-change > 1 and Q < 0.01. *Q* values represent the adjusted P values computed with the Benjamini & Hochberg method for controlling false discovery rate (FDR). c) Bubble plot of the depleted gene set in differentiated progeny of ∆SRR2-18^129/Cast^ vs. parent NSPCs generated by 10 days of *in vitro* differentiation.

**Figure S6. Chromatin accessibility differences in NSPCs following SRR2-18 deletion with ATAC-seq.** a) Normalized ATAC-seq read coverage at the transcription start site (TSS), gene body and flanking regions in parent and ΔSRR2-18^129/Cast^ NSPCs. b) Normalized ChIP-seq read coverage for histone post-translational modifications (H3K27ac, H3K4me1, H3K4me3) associated with regulatory DNA across parent NSPC ATAC-seq peaks. c) Bubble plot of the gene ontology terms enriched in the set of decreased accessibility regions in ΔSRR2-18^129/Cast^ vs. parent NSPCs. d) Bubble plot displaying enriched genomic regions in the set of increased accessibility regions in ΔSRR2-18^129/Cast^ vs. parent NSPCs. e) Normalized ATAC-seq and RNA-seq tracks over the *Hoxb* locus, displayed on the UCSC Genome Browser (mm10). Signal tracks show increased accessibility at the *Hoxb6-9* genes and increased total *Hoxb6-9* RNA in ΔSRR2-18^129/Cast^ NSPCs.

**Figure S7. Comparative analysis of chromatin accessibility patterns in differentiated and regionalized neural lineages.** a) Hierarchical cluster analysis of normalized counts from differential ATAC-seq peaks. Clustering revealed groups of genomic regions with differential accessibility that distinguished *Sox2*-mCherry tagged F1 NSPCs (parent) from ΔSRR2-18^129/Cast^, forebrain derived neural progenitors (NPC Forebrain), astrocyte, excitatory neurons (Neuron), and neural tube progenitors (NPC Neural Tube). b) GO biological process term enrichment across differentially accessible regions that define clusters 1-7. c) Principal component analysis of normalized ATAC-seq read counts showing that most datasets analyzed are well separated.

**Supplemental Tables**

**Table S1.** List of guide RNA (gRNA) target sequences

**Table S2.** List of primers for plasmid construction

**Table S3.** List of antibodies

**Table S4.** List of RT-qPCR primers

**Table S5.** Raw sequencing data compiled from the ENCODE consortium.

**Table S6.** Raw sequencing data compiled from NCBI Sequence Read Archive (SRA) and European Nucleotide Archive (ENA)

**Table S7.** Proximal Sox2 regulatory region coordinates.

**Table S8.** Differential gene expression analysis of in vitro differentiated parent NSPCs versus undifferentiated control.

**Table S9.** Differential gene expression analysis of SRR2-18 heterozygous deletion versus control.

**Table S10.** Gene set enrichment analysis for GO biological process terms in differentially abundant transcripts in SRR2-18 heterozygous deletion versus control.

**Table S11.** Differential gene expression analysis of SRR2-18 homozygous deletion versus control.

**Table S12.** Gene set enrichment analysis for GO biological process terms in differentially abundant transcripts in SRR2-18 homozygous deletion versus control.

**Table S13.** Differential gene expression analysis of differentiated progenies from SRR2-18 NSPCs homozygous deletion versus control.

**Table S14.** Gene set enrichment analysis for GO biological process terms in differentially abundant transcripts in differentiated progenies from SRR2-18 NSPCs homozygous deletion versus control.

**Table S15.** Differential chromatin accessibility analysis of SRR2-18 NSPCs homozygous deletion versus control.

**Table S16.** List of non-redundant SOX2 bound regions in neural progenitors from reanalyzed SOX2 ChIP-seq data.

**Table S17.** JASPAR motif matches recovered from intersectional SOX2 ChIP-seq, H3K27ac-modified and accessible chromatin regions in NSPCs.

**Table S18.** Genomic region enrichment analysis for GO biological process terms depleted in differentially accessible regions in SRR2-18 NSPCs homozygous deletion versus control.

**Table S19.** Genomic region enrichment analysis for GO biological process terms enriched in differentially accessible regions in SRR2-18 NSPCs homozygous deletion versus control.

**Table S20.** List of significant sequence-specific transcription factors in the Binding Analysis for Regulation of Transcription (BART) matching the ATAC-seq motif footprints.
