# Supplementary figures and images for: A Sox2 enhancer cluster regulates region-specific neural fates from mouse embryonic stem cells
Source: G3 (Bethesda). 2025 Jan 24;15(4):jkaf012. doi: 10.1093/g3journal/jkaf012 (PMC12005160; doi:10.1093/g3journal/jkaf012)

**a**

pGL4.23 Reporter  
Luminescence in NSPCs

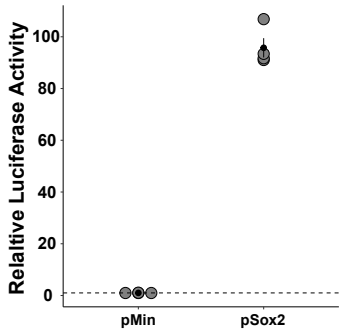**b**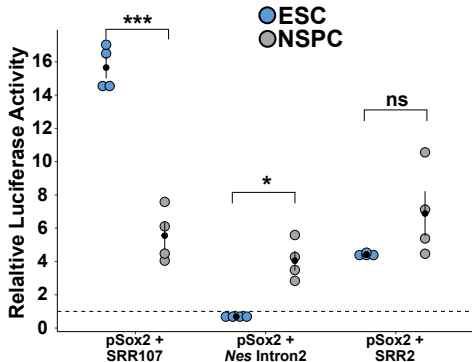

Supplement: jkaf012_Supplementary_Data [file jkaf012_supplementary_data.zip › Figure_S2_G3-2024-405518.pdf]

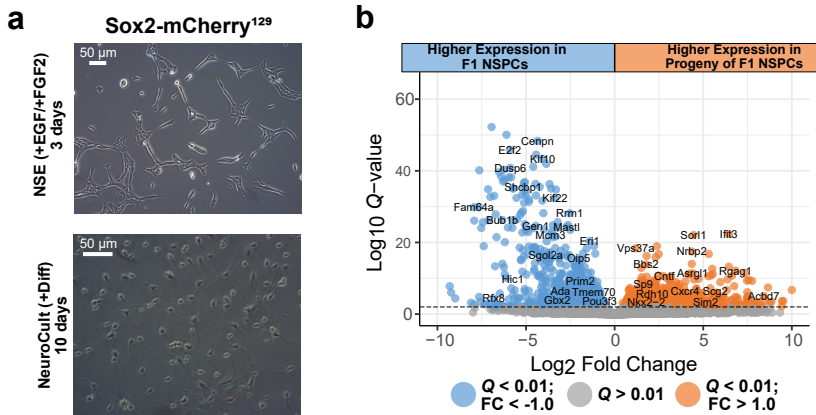

**c** **Allele-specific alignment file sorting with SNPsplitt**

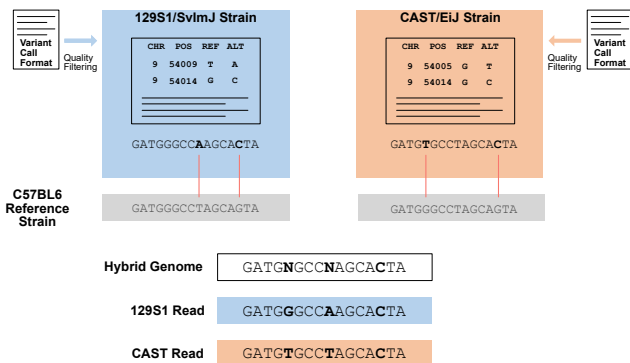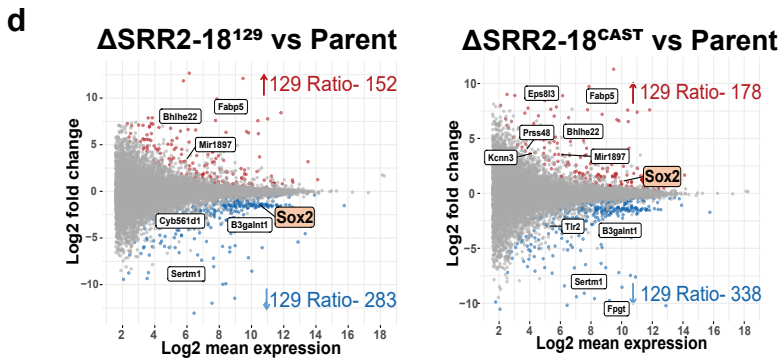

Supplement: jkaf012_Supplementary_Data [file jkaf012_supplementary_data.zip › Figure_S3_G3-2024-405518.pdf]

**a**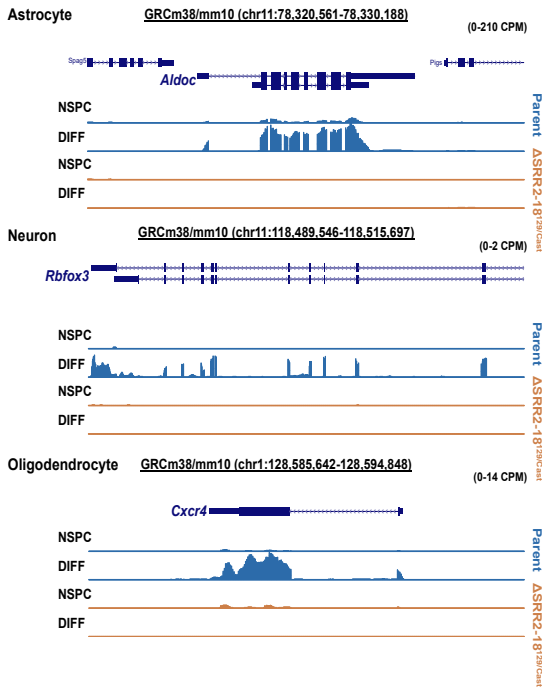**b**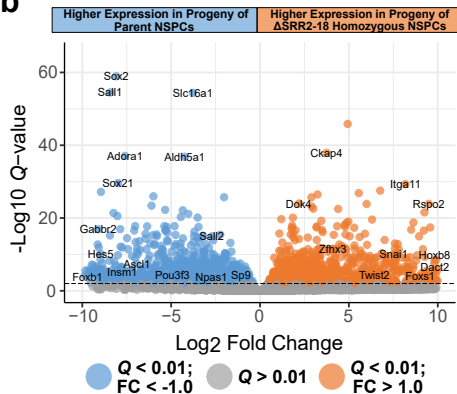**c**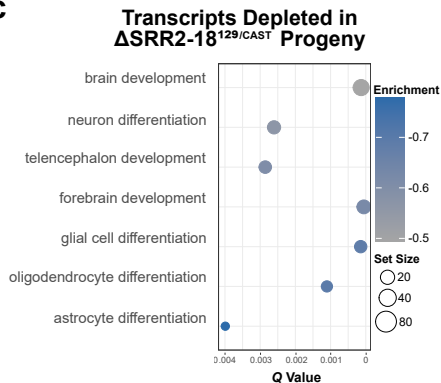

Supplement: jkaf012_Supplementary_Data [file jkaf012_supplementary_data.zip › Figure_S5_G3-2024-405518.pdf]
